# Hemidesmosome Mutations Contribute to the Onset and Severity of Acquired Autoimmune Bullous Diseases

**DOI:** 10.1002/mco2.70627

**Published:** 2026-02-15

**Authors:** Shan Cao, Tianyu Wang, Chen Lv, Shanshan Ma, Gongqi Yu, Qianqian Xia, Tingting Liu, Yueqian Yu, Lele Sun, Xiaoyan Pei, Qing Zhao, Zhenzhen Wang, Chuan Wang, Yongxia Liu, Shengli Chen, Jianwen Wang, Guizhi Zhou, Hong Liu, Yonghu Sun, Furen Zhang

**Affiliations:** ^1^ Dermatology Hospital of Shandong First Medical University Jinan China; ^2^ Shandong Provincial Institute of Dermatology and Venereology Shandong Academy of Medical Sciences Jinan China

**Keywords:** acquired autoimmune disorders, Pemphigoid, *Caenorhabditis elegans* model, genetic mutation, hemidesmosome, skin anchor junctions

## Abstract

Hemidesmosomes are structures that anchor junctions between basal epithelial cells and the basement membrane, essential for skin integrity. Genetic mutation of hemidesmosomes was well documented for the inherited bullous disorder, but is rarely investigated for acquired bullous disorders. We designed a 16‐gene targeted capture panel and sequenced 202 patients with hemidesmosomes‐related acquired disorders and 123 healthy controls, identifying 114 pathogenic variants in 15 genes, including 20.2% novel variants. Clinical relevance (disease severity and outcome) and immunohistochemistry results demonstrated that ITGA6, LAMC2, and EPPK1 mutations significantly affected the expression of hemidesmosome‐related proteins, compared with controls with non‐carriers. Functional studies in *Caenorhabditis elegans* models with transmission electron microscopy and confocal microscopy demonstrated that ITGA6 (ina‐1) mutation can disrupt the hemidesmosomes assembly network, such as cytolinker (vab‐10a) and apical (mup‐4) and basal (let‐805), thereby disrupting the hemidesmosome structure. This represents a quantitative to qualitative change in pemphigoid disease. Transcriptomic and serum proteomic analyses further revealed that ITGA6 mutations perturb epithelial development and hemidesmosome integrity, with both missense/loss‐of‐function variants leading to activation of  NOD‐like receptor–NF‐κB–TNF–pyroptosis signaling pathways. These findings highlight the critical role of hemidesmosome genetic variants in the development of not only inherited but also acquired autoimmune bullous disorders.

## Graphical abstract

This study examined hemidesmosome assembly‐related genes in pemphigoid diseases, revealing variants linked to disease onset and severity. Functional analyses, including *Caenorhabditis elegans* models, Ker‐CT transcriptomics, human proteomics, etc., demonstrated that ITGA6 mutations destabilize hemidesmosomes, disrupt dermal–epidermal adhesion, and increase susceptibility in patients, providing mechanistic insight into autoimmune targeting in pemphigoid disorders.

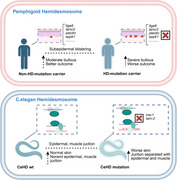

## 1 Introduction

Hemidesmosomes (HD) anchor the epidermal keratin filament cytoskeleton to the extracellular matrix, which is crucial for the mechanical integrity of skin [1, 2]. The core of each HD consists of at least five major components, including plectin (*PLEC*), bullous pemphigoid (BP) antigens *BP180* (type XVII collagen, BPAG2) and *BP230* (BPAG1e), tetraspanin *CD151*, and integrin a6b4, which interact with each other in multiple ways [2, 3, 4, 5, 6, 7, 8]. Genetic variations in any of these components can result in inherited bullous disorders termed epidermolysis bullosa (EB), which in some forms can be fatal [9]. Integrin α6β4 is a transmembrane receptor and a key component of the HD anchoring complex. Variants in *ITGA6* and *ITGB4* genes encoding integrin α6β4 compromise dermal–epidermal adhesion and are associated with autoimmune bullous and pyloric atresia [10, 11]. Recent studies have also identified pathogenic COL7A1 variants in patients with epidermolysis bullosa acquisita (EBA). As the major component of anchoring fibrils, COL7A1 is functionally distinct from core hemidesmosome assembly proteins, providing preliminary evidence that genetic mutations may increase susceptibility to pemphigoid diseases [12, 13]. Despite the well‐documented effect of pathogenic genetic variants on specific protein complexes in congenital EB, the role of pathogenic variants in acquired blistering diseases is largely unknown. A previous study found that although low *ITGA6* gene levels are sufficient for HD integrity, they are also associated with a mild skin phenotype in EB, an inherited bullous disorder [9, 12, 13, 14]. However, whether decreased *ITGA6* expression caused by genetic variants affects HD structural stability and induces acquired pemphigoid disease (PD) is unknown.

Autoantibodies against structural proteins of HD‐induced PD are characterized by tense blisters and erosions on the skin or mucous membranes, with the pathogenesis linked to the autoantibody presence [15, 16]. BP is the most common PD subtype [17]. Linear immunoglobulin A (LigA) dermatosis and dermatitis herpetiformis (DH) are the less common subtypes of PD than EBA [15, 18, 19, 20, 21]. The global incidence of BP is approximately 0.0419 per 1000 person‐years, with an average clinical prevalence of approximately 0.79% [17]. Immunoglobulin G (IgG) deposition in the dermoepidermal junction is a common pathological feature of most PD subtypes [15, 22, 23, 24, 25]. That is why intravenous administration of high‐dose IgG (IVIG) has been shown to be effective in patients with steroid‐resistant BP in clinical practice, which suggests that the inhibitory effects of IVIG on BP are associated with the reduction in pathogenic IgG level and cytokine production modulation [25].

There has been limited recent success in identifying genetic associations between PD and HLA allelotypes, such as HLA‐DQB1*03:01 with BP [26], HLA‐B*08:01 with DH [27], HLA‐DRB1*15:03 with EBA [28], and HLA‐DQB1*02:01 with LigA [29]. Very few studies have investigated the role of candidate genes across the PD spectrum. In addition, genetic desmosome variants, including desmoglein (Dsg) 1–4, desmocollin 1–3, tuftelin 1, desmosomal gene desmoplakin, and plakophilin 1–3, a type of intercellular junction found in epithelial cells, have been reported to result in a variety of erosive skin and mucosal phenotypes and other nonskin diseases, such as heart disorders (dilated cardiomyopathy) [30, 31, 32, 33, 34].

In the present study, it was hypothesized that rare pathological variants in *ITGA6*, an HD assembly‐related gene, might affect HD structural stability, thereby aggravating epidermal dermis junction separation in acquired autoimmune bullous disease pathogenesis.

## 2 Results

### 2.1 HD Assembly‐Related 16‐Gene Variant Panel in the PD Spectrum

**FIGURE 1:**
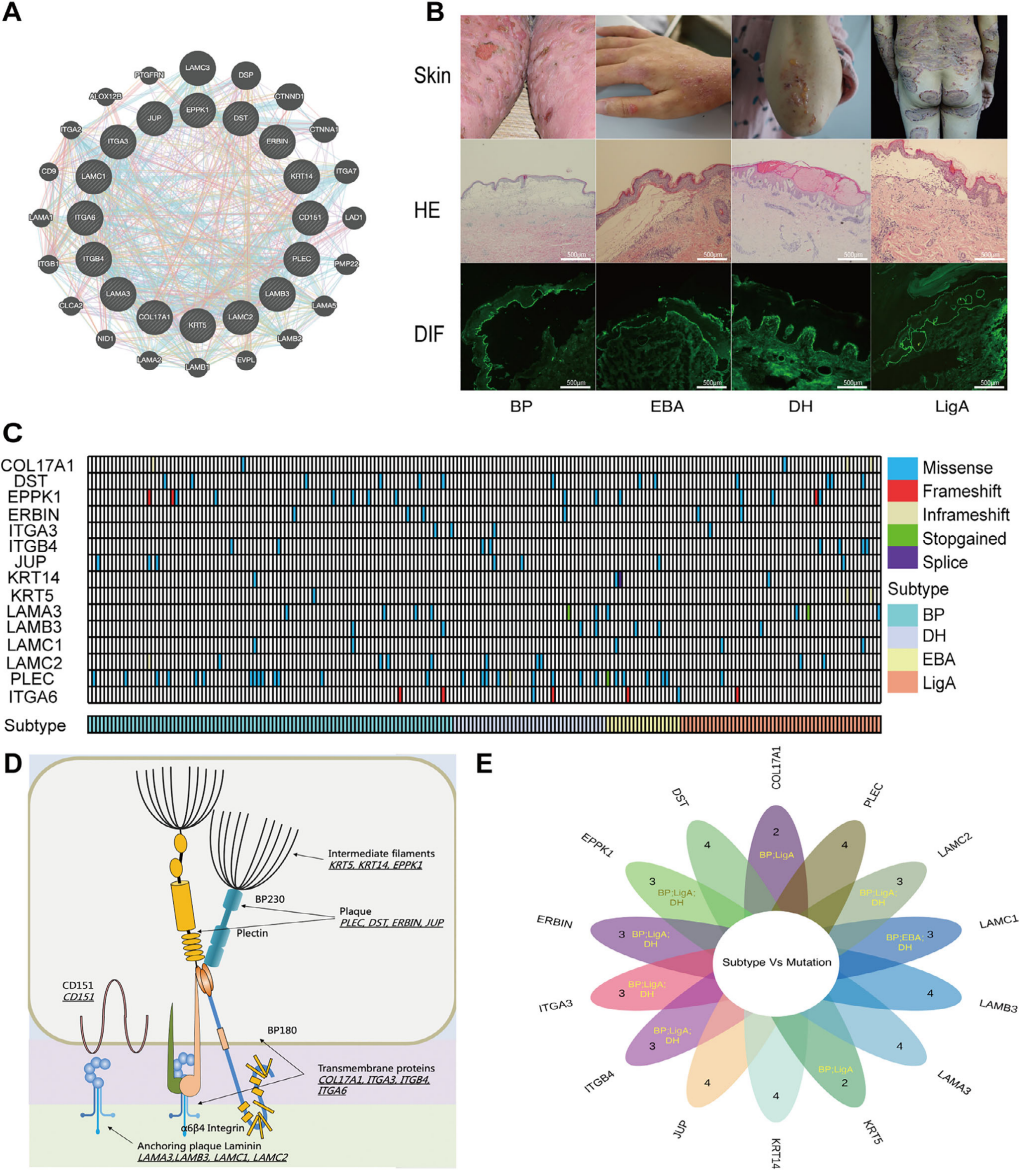
Diagram of hemidesmosome assembly‐associated genes and mutation distribution in each PD subtype. (A) Hemidesmosome assembly‐associated 16‐gene network constructed using GO and GeneMANIA. (B) Clinical, histological, and direct immunofluorescence of PD subtype characteristics. (C) Mean depth, capture panel coverage rate, and mutation distribution in each gene and PD subtype. (D) Diagram of the hemidesmosome and location of each gene. (E) Shared mutations by each PD subtype.

A next‐generation sequencing‐based gene panel was designed and included 16 HD assembly‐related genes (*CD151, COL17A1, DST, EPPK1, ERBIN, ITGA3, ITGA6, ITGB4, JUP, KRT14, KRT5, LAMA3, LAMB3, LAMC1, LAMC2*, and *PLEC*) based on the Gene Ontology (GO) enrichment analysis (GO:0031581, Figure 1A). A total of 202 PD patients who met the diagnosis criteria were recruited in the study, including 93 BP, 18 EBA, 51 LigA, and 40 DH patients (Figure 1B; Tables S1, S2). All of the patients and 123 healthy control individuals were genotyped using the HD assembly‐related 16‐gene panel. Overall, the mean target coverage for these HD‐related genes was 1102×, ranging from 614× to 1542×. The mean coverage rate for the 16 designed regions was 96.9%, ranging from 95.2% to 98% (Figure 1C).

A total of 114 pathogenic variants fulfilling the pathogenicity criteria (Table 1) were identified within 15 genes, where 20.2% (*n* = 23) of variants were novel (Figure S1). The *CD151* gene did not meet the pathogenicity criteria and was excluded. All variants were heterozygotes and not found in 123 healthy controls. Nine insertion–deletion variants were those of frameshift elongation or truncation, in‐frame insertion or deletion, and disruptive in‐frame deletion, such that 44.4% (*n* = 4) were novel (p.R37fs of *COL17A1*, p.D2407delinsPD, and p.T2405fs of *EPPK1*, and p.A765fs of *LAMC2*). In addition, 18 novel missense variants were identified. The variant genes allocated to the HD components included intermediate filaments, plaque proteins (*PLEC* and *BP230*), transmembrane proteins (integrin α6β4 and *BP180*), and anchoring plaque laminin, but were negative for tetraspanin *CD151* (Figure 1D).

**TABLE 1:** Genetic variants identified in 15 hemidesmosome assembly genes in 202 individuals with pemphigoid disease.

Gene	Chr	Nucleotide	Protein	SNP accession number	Functional effect	PD phenotype
*ITGA6*	3	c.A5757C	p.G233A	Novel	Missense	DH;EBA
	10	c.7540_7541insA	p.A249G	rs566695492	Inframe del	BP;LigA;EBA;DH
*COL17A1*	5	c.C316T	p.R106C	rs146267259	Missense	BP
	39	c.G2680A	p.G894R	rs765873070	Missense	LigA
	45	c.3000_3008del	p.1000_1003del	rs753169679	Inframe del	BP;LigA
*DST*	4	c.G317T	p.R106L	rs780894269	Missense	DH
	12	c.C1781G	p.A594G	rs1212844182	Missense	LigA
	23	c.A4772C	p.Q1591P	rs138185589	Missense	BP
	23	c.A4100G	p.Q1367R	Novel	Missense	EBA
	23	c.A3794T	p.D1265V	Novel	Missense	BP
	23	c.C3589T	p.R1197C	rs147377638	Missense	BP
	27	c.G3826A	p.E1276K	rs1435348308	Missense	BP
	31	c.A5147C	p.Q1716P	rs1376171798	Missense	LigA
	47	c.A9653T	p.H3218L	rs758568001	Missense	LigA
	47	c.G9517A	p.D3173N	rs186699708	Missense	BP
	56	c.G11161A	p.D3721N	rs41271862	Missense	EBA
	77	c.G14468A	p.R4823H	rs768427386	Missense	LigA
*EPPK1*	2	c.C6715T	p.R2239C	rs527298231	Missense	LigA
	2	c.G6495T	p.Q2165H	rs373872149	Missense	BP
	2	c.C6268T	p.R2090W	rs782111553	Missense	BP
	2	c.G5806A	p.A1936T	rs371992082	Missense	BP
	2	c.G4942A	p.A1648T	rs370754670	Missense	DH
	2	c.C4495T	p.R1499W	rs782093131	Missense	BP;LigA
	2	c.C4223T	p.A1408V	rs368940625	Missense	BP
	2	c.C2233T	p.R745W	rs782758450	Missense	DH
	2	c.G2212A	p.V738M	rs746589906	Missense	BP
	2	c.108dupC	p.R37fs	Novel	Frameshift	BP
	2	c.G7121A	p.R2374H	rs782512704	Missense	LigA
	2	c.G6653T	p.R2218L	Novel	Missense	DH
	3	c.A7213G	p.T2405A	rs781881077	Missense	BP;LigA
	3	c.7218_7219insCCC	p.D2407delinsPD	Novel	Inframe ins	BP;LigA
	3	c.7214_7215insTG	p.T2405fs	Novel	Frameshift	BP;LigA
	3	c.7205_7209del	p.Q2402fs	rs145250036	Frameshift	BP;LigA
*ERBIN*	3	c.A121G	p.I41V	Novel	Missense	LigA
	21	c.A3320G	p.Y1107C	rs201110697	Missense	BP;LigA
	22	c.T3658C	p.S1220P	rs573605829	Missense	BP;BP
	22	c.G3795T	p.Q1265H	rs759126759	Missense	DH
*ITGA3*	4	c.T487C	p.Y163H	Novel	Missense	DH
	7	c.G1016A	p.G339E	Novel	Missense	BP
	12	c.C1570G	p.R524G	rs566775051	Missense	LigA
	22	c.G2795A	p.R932Q	rs781502548	Missense	BP
*ITGB4*	5	c.C491T	p.T164I	rs1483189038	Missense	BP
	16	c.C2020T	p.R674W	rs1191842048	Missense	LigA
	21	c.A2554G	p.N852D	Novel	Missense	DH
	27	c.G3385A	p.A1129T	rs201321412	Missense	LigA
	28	c.C3551T	p.T1184I	Novel	Missense	LigA
	31	c.G4003A	p.V1335M	rs1398068301	Missense	LigA
	31	c.C4063T	p.R1355C	rs530031041	Missense	BP
	36	c.C4916T	p.S1639L	rs370378040	Missense	DH
*JUP*	3	c.G457A	p.D153N	rs368479692	Missense	LigA
	3	c.T449G	p.L150R	Novel	Missense	EBA
	4	c.C560T	p.A187V	rs782370709	Missense	DH
	6	c.C958T	p.R320C	rs200740462	Missense	BP
	11	c.T1862C	p.I621T	rs752594411	Missense	DH
	11	c.G1807T	p.V603L	rs200327969	Missense	BP
*KRT14*	1	c.G525A	p.K175K	rs768714133	Splice site	DH
	4	c.G896A	p.R299H	rs199868373	Missense	BP;EBA
	6	c.C1186G	p.Q396E	rs58393329	Missense	LigA
*KRT5*	1	c.G8A	p.R3H	rs770091254	Missense	BP
	9	c.1550_1573del	p.517_525del	rs577328983	Inframe del	LigA
*LAMA3*	5	c.G851A	p.R284Q	rs991127113	Missense	LigA
	6	c.T628C	p.C210R	Novel	Missense	BP
	17	c.G2057A	p.R686H	rs199546528	Missense	BP;EBA
	19	c.C2233T	p.R745Ter	rs192156286	Nonsense	DH;LigA
	28	c.C3586T	p.R1196W	rs750139027	Missense	BP;LigA
	32	c.C3995T	p.T1332M	rs745375536	Missense	DH
	36	c.C4796G	p.T1599R	Novel	Missense	BP
*LAMB3*	11	c.C1348T	p.R450C	rs200895463	Missense	BP;EBA
	17	c.C2605T	p.R869C	rs11555728	Missense	DH
	20	c.C3124T	p.R1042W	rs114040223	Missense	LigA
	21	c.T3368A	p.M1123K	rs78483218	Missense	BP;DH
	21	c.G3311A	p.R1104Q	rs149573831	Missense	EBA
*LAMC1*	2	c.C466A	p.R156S	Novel	Missense	LigA
	4	c.C962T	p.P321L	Novel	Missense	BP;EBA
	14	c.C2587T	p.R863W	rs773515901	Missense	LigA
	19	c.G3347A	p.R1116H	rs548688323	Missense	BP
*LAMC2*	3	c.C371T	p.T124M	rs11586699	Missense	DH;DH
	4	c.C460T	p.R154C	rs535636160	Missense	BP
	5	c.A539G	p.N180S	COSV51458912	Missense	LigA
	8	c.C985T	p.P329S	Novel	Missense	BP
	15	c.2294_2300del	p.A765fs	Novel	Dis inframe del	BP
	16	c.T2333A	p.L778Q	rs1342054556	Missense	DH
	17	c.C2569T	p.R857W	rs780621237	Missense	BP
	20	c.G3031A	p.A1011T	rs201820165	Missense	LigA
*PLEC*	1	c.C100T	p.Q34Ter	rs781805561	Nonsense	EBA
	2	c.G10A	p.E4K	Novel	Missense	DH
	20	c.G2266A	p.E756K	rs369133057	Missense	DH
	26	c.G3245A	p.R1082H	rs375617818	Missense	BP
	27	c.C3595T	p.R1199C	rs1369858558	Missense	BP
	27	c.G3547A	p.V1183M	rs3134600	Missense	BP
	30	c.C3916T	p.R1306C	rs372256096	Missense	DH
	31	c.G7151A	p.R2384H	rs368343687	Missense	BP
	31	c.A6674C	p.E2225A	Novel	Missense	BP
	31	c.C6196T	p.R2066C	rs868963575	Missense	BP
	31	c.C5806T	p.R1936C	rs566539439	Missense	BP
	31	c.A5585G	p.Q1862R	Novel	Missense	DH
	31	c.C5465T	p.A1822V	rs782216341	Missense	DH
	31	c.C5429T	p.A1810V	rs542642242	Missense	BP
	31	c.G5412T	p.Q1804H	Novel	Missense	EBA
	31	c.C4270T	p.R1424W	rs531928668	Missense	DH
	32	c.C13505T	p.T4502I	rs1320878270	Missense	BP
	32	c.C13198T	p.R4400C	rs200447944	Missense	BP
	32	c.G12739A	p.A4247T	rs200924154	Missense	EBA;DH
	32	c.G12605A	p.R4202H	rs151106439	Missense	BP;EBA;DH
	32	c.G12341A	p.R4114Q	rs533757341	Missense	EBA
	32	c.G11035A	p.A3679T	rs369497741	Missense	DH
	32	c.C10888T	p.R3630C	rs781932676	Missense	BP
	32	c.G10760A	p.R3587Q	rs369570610	Missense	BP
	32	c.C10174T	p.R3392C	rs781882032	Missense	LigA
	32	c.G9997A	p.V3333M	rs782748769	Missense	BP
	32	c.C8890T	p.R2964C	rs782297488	Missense	LigA
	32	c.G8567A	p.R2856H	rs781845486	Missense	DH
	32	c.C8353T	p.R2785W	rs561686703	Missense	BP;EBA
	32	c.7726_7728del	p.2576_2576del	rs782142905	Inframe del	DH

Abbreviations: PD: pemphigoid disease; DH: dermatitis herpetiformis; EBA: epidermolysis bullosa acquisita; BP: bullous pemphigoid; LigA: linear IgA bullous dermatosis.

A total of 139 pathogenic variants were identified in 119/202 (58.9%) PD patients. Stratified analysis by PD subtype showed different variant proportions: 47.3% of BP (*n* = 44), 77.8% (*n* = 14) of EBA, 70.0% (*n* = 28) of DH, and 64.7% (*n* = 33) of LigA (Figure 1E).

### 2.2 Clinical Relevance of the HD Assembly‐Related Gene Mutation

**FIGURE 2:**
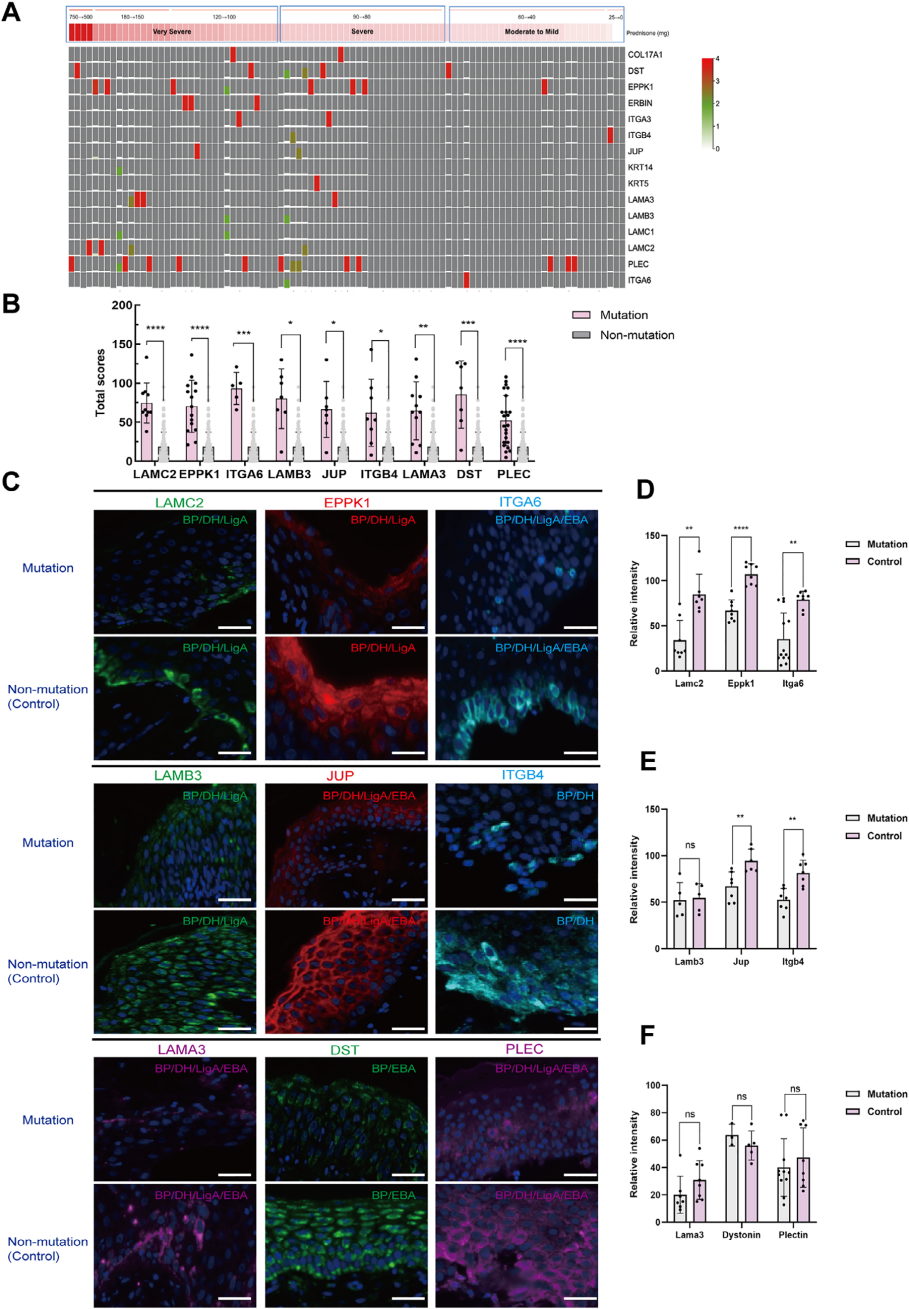
Clinical disease severity and outcome score are associated with hemidesmosome assembly‐related gene variation. (A) Correlation between variant counts and the maximum prednisone‐equivalent dose in 93 BP patients. (B) Positive correlation between mutation (*n* = 93) and control (*n* = 106) PD patients with nine hemidesmosome assembly‐related genes. (C) Representative immunohistochemistry images of PD patients carrying mutations and controls. Scale bar: 75 µm. (D–F) Quantitative analysis of immunohistochemistry results between mutations (*n* = 67) and controls (*n* = 62). ^*^ *p* ≤ 0.05, ^**^ *p* ≤ 0.01, ^***^ *p* ≤ 0.001, and ^****^ *p* ≤ 0.0001. *p‐*values are calculated using a two‐tailed Student's *t*‐test.

To investigate the potential pathogenic effect on all 202 PD patients, medical records were reviewed to retrieve clinical variables, such as disease severity and outcome. The correlation between the clinical index and variant counts was then analyzed. Ninety‐three BP patients were stratified according to disease severity. The “very severe” group had the highest mutation rate of 65.7% (23/35), the “severe” group showed a mutation rate of 53.6% (15/28), and the “mild to moderate” group had a rate of 20% (6/30), suggesting a positive correlation between disease severity and pathogenic mutations (Figure 2A).

In addition, the calculation of the total score is equal to the sum of the scores obtained from three aspects: the IIF score, the cumulative duration of medication (in months), and the disease outcome (which includes three categories: remission, on maintenance therapy, and death). Mutation burden and disease association scores were significantly higher in variant carriers (n = 93) than in non‐carriers (n = 106) among BP patients. Gene‐based analyses further identified nine genes significantly associated with disease severity/outcome, including *LAMC2* (*p* < 0.0001), *EPPK1* (*p* < 0.0001), *ITGA6* (*p* = 0.0007), *LAMB3* (*p* = 0.0112), *JUP* (*p* = 0.0231), *ITGB4* (*p* = 0.0384), *LAMA3* (*p* = 0.0059), *DST* (*p* = 0.0059), and *PLEC* (*p* < 0.0001) in Figure 2B and Table S3.

Furthermore, the effect of variants on protein expression and HD assembly‐related gene function was assessed using immunohistochemistry (IHC) experiments. The results showed significantly reduced expression levels of relevant proteins in PD patients expressing variant proteins localized to the epidermis, epidermal‐dermal junction, and dermis (Figure 2C). Quantitative IHC analysis of cutaneous protein expression demonstrated a statistically significant difference (*p* = 0.02) between variant carriers (*n* = 67) and noncarriers (*n* = 62) for proteins encoded by putative pathogenic genes, including *LAMC2*, *EPPK1*, *ITGA6*, *JUP*, and *ITGB4* genes (Figure 2D–F).

### 2.3 Pathogenic Mutations in HD Assembly‐Related Genes with PD Phenotype Decrease Skin‐Related Protein Expression

**FIGURE 3:**
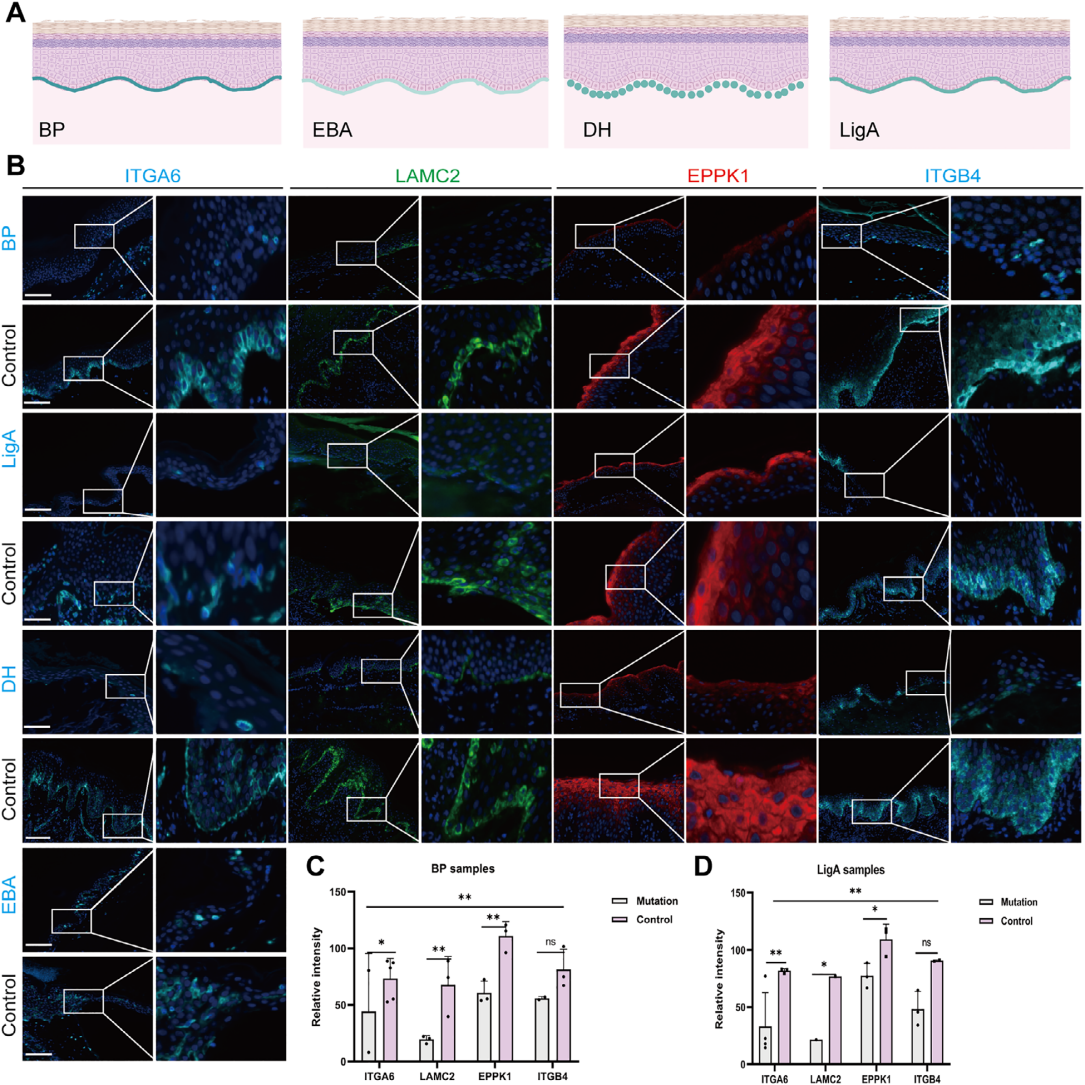
Pathogenic variants in hemidesmosome assembly‐related genes in pemphigoid disease spectrum decrease skin‐related protein expression. (A) Pattern diagram showing the location and pattern of pathogenic antibody deposition for different PD subtypes. (B) Representative immunohistochemistry images of BP, LigA, DH, and EBA mutation‐carrying and control patient samples stained for *ITGA6*, *LAMC2*, *EPPK1*, and *ITGB4*, *n* = 10, 14 for mutations and controls. Scale bar: 150 µm. (C) Quantitative analysis of IHC results for BP samples. (D) Quantitative analysis of IHC results for LigA samples, *n* = 11, 11 for mutations and controls. *p‐*values are calculated using a two‐tailed Student's *t*‐test. ^*^ *p* ≤ 0.05, ^**^ *p* ≤ 0.01. Each sample is represented as one dot.

The location and pattern of pathogenic IgG and/or C3 or IgA antibody deposition with different PD spectrums are represented on a model diagram (Figure 3A). To further verify the protein expression dysfunction caused by the pathogenic mutation of HD assembly‐related genes with PD spectrum, nonvariant PD controls (*n* = 35, including random 14 BP, nine LigA, seven DH, and five EBA patients) and variant carriers (*n* = 34, including random 10 BP, 11 LigA, 10 DH, and three EBA patients) were constructed using IHC experiment data. Scanning images of tissue samples were obtained using the Akoya Phenocycler Fusion (AKOYA Biosciences, USA). Skin protein expression levels of *ITGA6*, *LAMC2*, *EPPK1*, and *ITGB4* were significantly decreased in the mutant group when compared with those in the nonvariant controls. Next, quantitative protein analyses of variant carriers versus noncarrier PD controls for different PD phenotypes were performed to evaluate the influence of the above mutant genes on skin protein expression. As a result, *ITGA6*, *LAMC2*, and *EPPK1* all showed significantly decreased protein expression in BP and LigA skin lesions (Figure 3B,C). The protein expression level of the mutated *ITGB4* gene was not statistically significantly different compared with that of the nonvariant controls. DH and EBA were not analyzed due to the small sample size.

Overall, IHC and statistical analyses results further confirmed that *ITGA6*, *LAMC2*, and *EPPK1* mutations significantly affected the expression of skin‐related proteins, demonstrating statistically significant differences compared with controls with non‐carriers.

### 2.4 HD‐related Gene *Ina‐1* Mutation Affects Protein Expression in *Caenorhabditis elegans* Models

**FIGURE 4:**
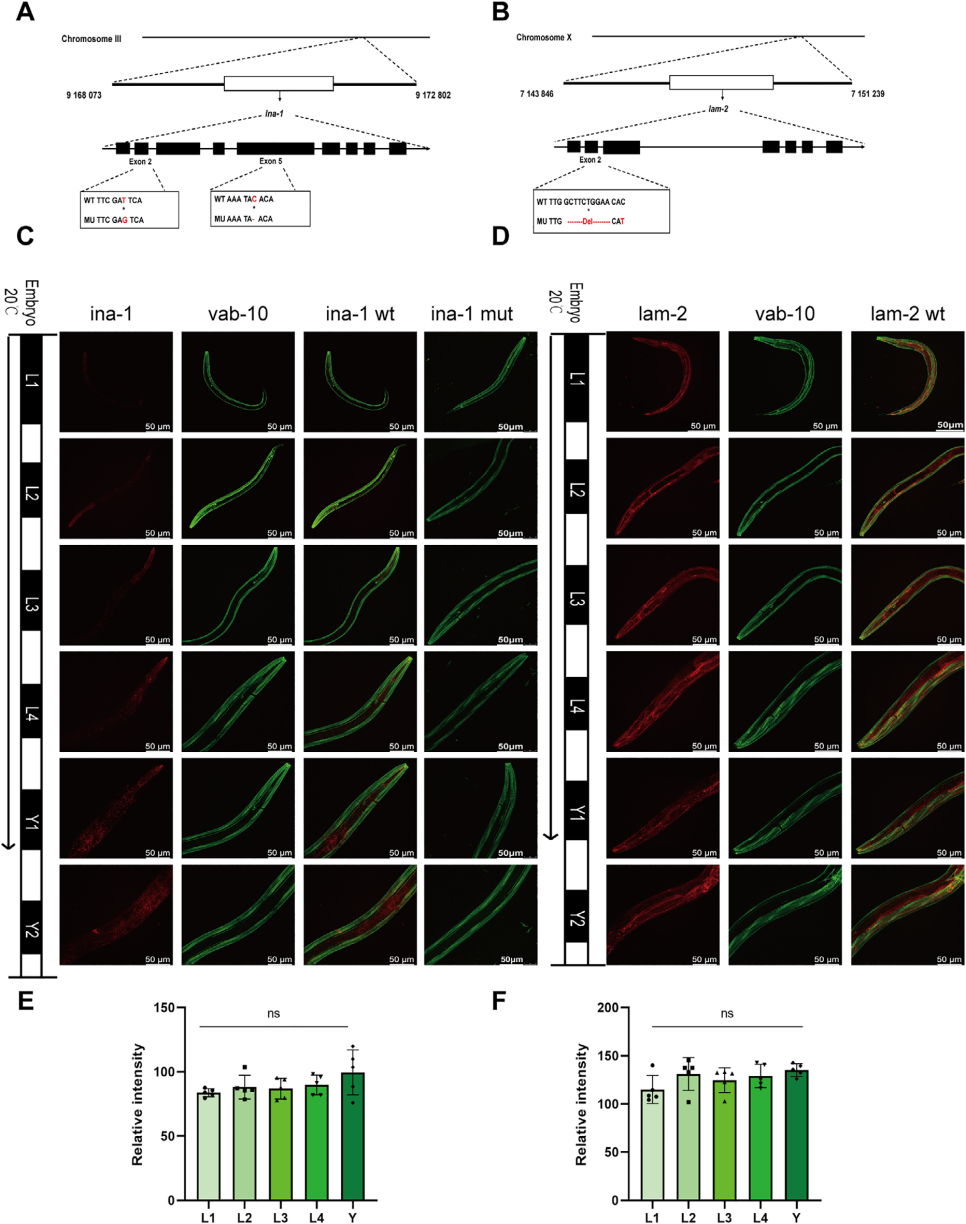
Hemidesmosome‐related gene *ITGA6* homologue *ina‐1* mutation affects protein expression in *C. elegans* models. (A) Diagram of *ina‐1* mutation pattern in *C. elegans* models and positions of residues mutated by CRISPR‐Cas technology. (B) Diagram of the *lam‐2* mutation pattern in *C. elegans* models and positions of residues mutated by CRISPR‐Cas technology. (C) Representative images of homozygous *ina‐1* wild‐type and mutation type embryos for CRISPR‐Cas knock‐in *ina‐1*::wrmScarlet and *vab‐10a::GFP* with the developmental process from L1, L2, L3, and L4 larvae to adult indicated on the left and right, respectively. Scale bar: 50 µm. Panels 1–4 show wrmScarlet, GFP, and merged signals for the turn of wild‐type *C. elegans* from L1–L4 larvae to adult (Y1 and Y2 are both adult locations), respectively. (D) Representative images of homozygous embryos for CRISPR‐Cas knock‐in *lam‐2*::wrmScarlet and *vab‐10a::GFP* with the developmental process from L1–L4 larvae to adult. Scale bar: 50 µm. Each sample is represented as one dot. (E) Quantitative analysis of confocal microscopy results for *ina‐1*::wrmScarlet *C. elegans* samples shows stable expression of fluorescence signals from L1–L4 larvae to adult. (F) Quantitative analysis of confocal microscopy results for *lam‐2*::wrmScarlet mutants shows stable expression of fluorescence signals from L1–L4 larvae to adults.

*Caenorhabditis elegans* provides a robust model to investigate HD structure assembly, since *C. elegans* HD structures (CeHDs) have the morphological hallmarks of vertebrate HD. To investigate whether the *ITGA6* and *LAMC2* pathogenic variants observed in PD patients affect HD structural integrity, wild‐type and mutant *ina‐1* (*ITGA6*) and *lam‐2* (*LAMC2*) *C. elegans* models were constructed to explore the study hypothesis using CeHDs (Figure 4A,B). Vab‐10 generates isoforms related either to *PLEC* (termed *VAB‐10A*) or to microtubule actin cross‐linking factor plakins (termed *VAB‐10B*). The *Vab‐10a*::green fluorescent protein (GFP) is a fusion protein created by fusing GFP to the *Vab‐10* gene to visualize the location of the *Vab‐10a* protein in live cells. *Vab‐10a*::GFP protein in CeHDs presented as circumferential bands in the L1–4 larval stages and adult epidermis and as HD structure in the PD spectrum.

The *ina‐1*::wrmScarlet is a fusion protein created with red fluorescent protein (wrmScarlet) to the *ina‐1* gene. The *ina‐1*::wrmScarlet protein in CeHDs was present at the junction between epidermis and muscle on confocal images. Mutation of the *ina‐1* gene caused a complete loss of the striped CeHDs pattern in the epidermis compared with that in the wild‐type samples (Figure 4C). Most of the *lam‐2* protein expression was observed close to the muscles (Figure 4D). Furthermore, *lam‐2 C. elegans* mutants were not apparent on confocal microscopy images due to the homozygous lethality. The relative intensity analysis of *lam‐2*::wrmScarlet mutants showed stable expression of fluorescence signals from L1–4 larvae to adults (Figure 4E,F).

Overall, the corresponding *ina‐1 C. elegans* models were successfully constructed based on *ITGA6* gene mutations in PD patients. In addition, *ina‐1*::GFP fluorescence expression decreased after mutation and changes in the expression of cytolinker *vab‐10a* in CeHDs.

### 2.5 The *Ina‐1* Mutation Mediated Junction Separation of Skin Structures in *C. elegans* Models

**FIGURE 5:**
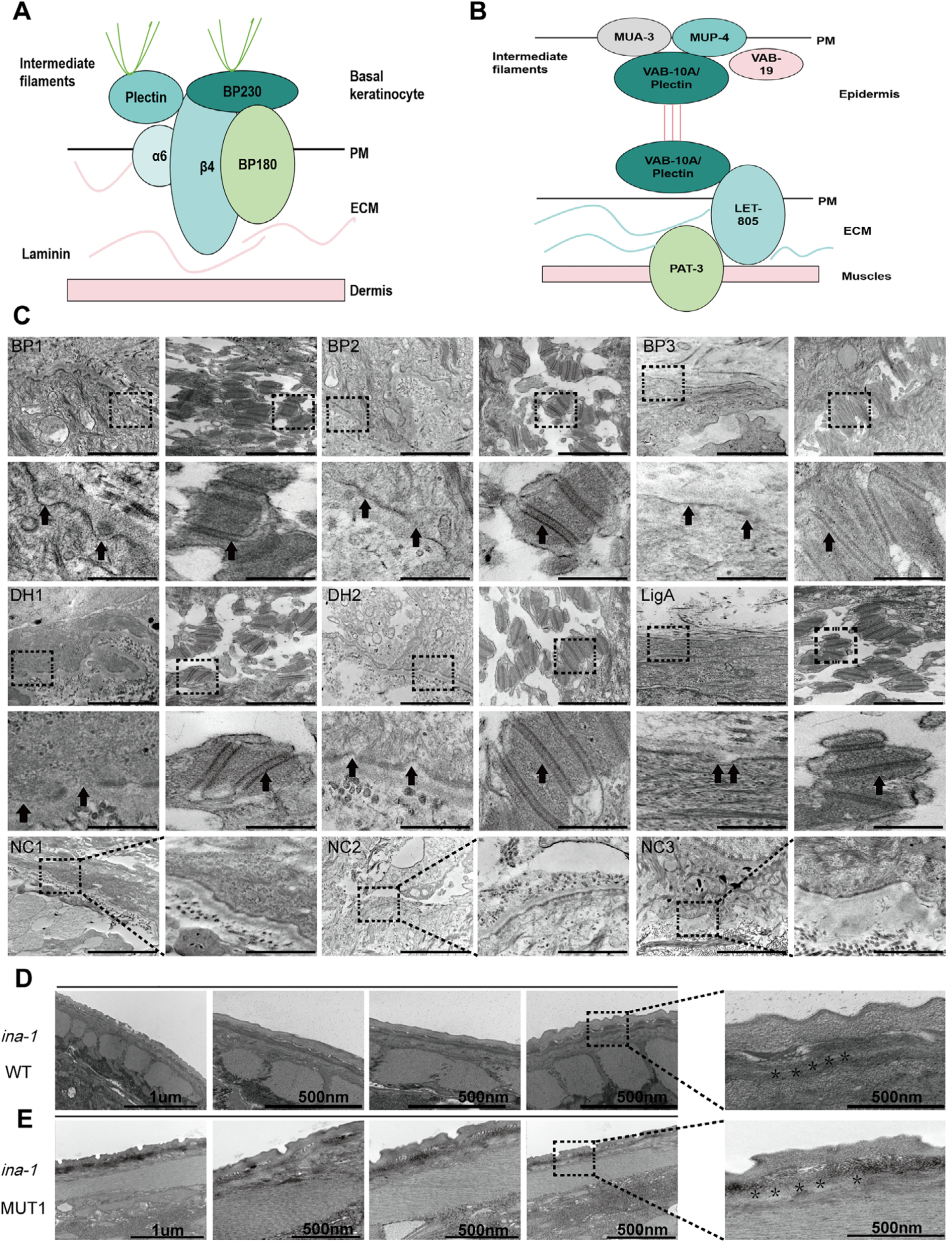
*ITGA6* homologue *ina‐1* mutation mediated the PD‐like phenotype in *C. elegans* models. (A) Components and structures of mammalian hemidesmosome type in the skin of PD patients. (B) Components and structures of CeHDs in epidermis adjacent to body‐wall muscles. (C) Transmission electron microscopy (TEM) results revealed that hemidesmosome structures were destroyed in patients with different PD forms (*n* = 6, including BP = 3, DH = 2, LigA = 1) and healthy controls (*n* = 3). Desmosome structures were normal and served as controls. Black arrows indicate abnormal hemidesmosome structures and normal desmosome structures. Pat 2 represents high magnifications of the region highlighted by dashed rectangles in BP1, BP2, BP3, DH1, DH2, and LigA1. Scale bar: 500 nm. (D) Representative image of *ina‐1* wild‐type (WT) shows clear epidermal and muscle junction structure border and normal cuticle basal layer in *C. elegans*. Scale bar: 1 µm. Scale bar: 500 nm. (E) Epidermal and muscle structure border and basal cuticle layer were basically normal with mild separation phenomena in the *ina‐1* type (MUT1). Scale bar: 1 µm; 500 nm.

To verify that *ina‐1* mutants affecting CeHDs could be used as a model to investigate HD structures in the PD spectrum, transmission electron microscopy (TEM) was used to observe whether a comparable phenotype occurred between PD patients and *C. elegans* models. Components and structures of mammalian‐type HD in the skin of PD patients and CeHDs in *C. elegans* models were observed (Figure 5A,B). Inadequate HD numbers and density in ∼90% of observed fields along the epidermal basement membrane zone were observed in PD. TEM images revealed fewer, lower quality, smaller‐sized HD structures in six PD patient skin lesion samples (*n* = 6, including three BP cases, two DH cases, one LigA case) and three healthy controls). No dermal–epidermal separation was observed; the basal lamina remained attached to basal keratinocytes. The desmosome structures were normal and served as the controls to exclude the effect of skin sampling (Figure 5C). Wild‐type *ina‐1* showed a clear border between the epidermal and muscle junction structure and a normal basal cuticle layer, and the neighboring normal muscle (Figure 5D). The epidermal and muscle structure border was blurred in *ina‐1* mutants, while the basal cuticle layer showed minor degradation in *ina‐1* mutants compared with wild‐type samples (Figure 5E).

Collectively, these results suggested that *ina‐1 C. elegans* mutants could serve as a good model for investigating HD structures in PD patients with *ITGA6* mutation.

### 2.6 Loss of *Ina‐1* Function Affects CeHDs Integrity During *C. elegans* Embryonic Development

**FIGURE 6:**
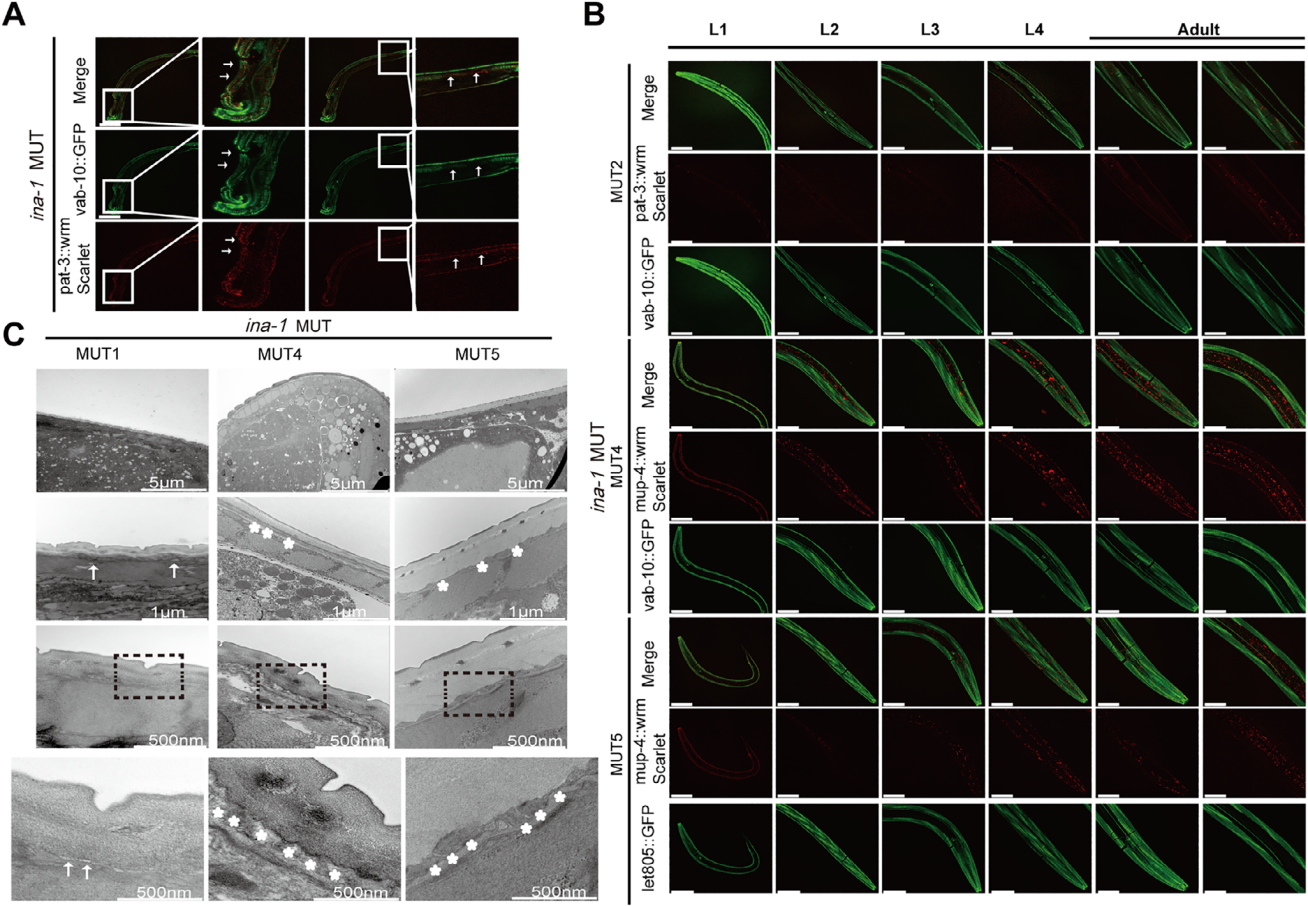
Loss of *ina‐1* function affects CeHDs’ integrity during embryonic development. (A) Severe phenotypic destruction in *C. elegans* with the *ina‐1* mutation without the qC1 balancer. White arrows indicate head defects, and dense plaques in the distal part of the body are severely damaged. Pat 2 represents high magnifications of the region highlighted by dashed rectangles in the *ina‐1* mutation. (B) Confocal microscope images show immunofluorescence of MUT2, MUT4, and MUT5 from L1, L2, L3, and L4 larvae to adults in *C. elegans*. Immunofluorescence analysis of cytolinker (green, *vab‐10a*), apical (red, *mup‐4*), and basal (green, *let‐805*) CeHDs, and muscles (red, *pat‐3*) in L1–4 larvae and adults. Scale bar: 50 µm. (C) TEM micrographs of the adult mutant body wall in the epidermal and muscle junction structure region. Gaps indicated by arrows and asterisks were observed between apical hypodermis and basal cuticle, especially in MUT4 and MUT5 retrospective images, compared with MUT1. The hypodermis remained tightly attached to the muscle in the detachment region. More asterisks mean larger gaps and more separation. Scale bar: 5 µm, 1 µm, and 500 nm.

The underlying mechanism of junction separation in *ina‐1* mutant *C. elegans* skin structure was further explored. Confocal microscopy and TEM were utilized to observe whether CeHDs integrity was affected in *ina‐1* mutants. Homozygous *ina‐1* mutant *C. elegans* showed body morphology defects with severe phenotypic destruction in the head of *C. elegans*. Head defects and dense plaques in the distal part of the body were observed along with significantly decreased *vab‐10a* and *pat‐3* expression levels (Figure 6A).

To further investigate *ina‐1* mutants, the balancers qC1[dpy‐19(e1259) glp‐1(q339)] were added to maintain deleterious mutations, prevent recombination, and follow chromosomes. Confocal microscopy images were used to perform immunofluorescence comparisons with multimutants MUT2, MUT4, and MUT5. Immunofluorescence analysis of cytolinker (*vab‐10a*::GFP), apical (*mup‐4*::wrmScarlet), and basal (*let‐805*::GFP) CeHDs, and muscles (*pat‐3*::wrmScarlet) in L1–4 larvae and adults showed *vab‐10a* in CeHDs as circumferential bands in MUT2 and MUT4. However, the expression of apical (*mup‐4*::wrmScarlet) and basal (*let‐805*::GFP) CeHDs significantly decreased in MUT5 compared with MUT4 from L1–4 larvae and adults (Figure 6B). TEM micrographs of the adult mutant body wall in the region of epidermal and muscle junction structure were utilized to observe changes in the phenotype of MUT1, MUT4, and MUT5. Interestingly, larger gaps were observed between the apical hypodermis and basal cuticle, especially in MUT4 and retrospective MUT5 images compared with those of MUT1, although the hypodermis remained tightly attached to the muscle in the detachment region (Figure 6C).

The observed phenotypic changes supported the study hypothesis that the junction separation with skin structure in ina‐1 mutated *C. elegans* was related to the assembled CeHDs components. In summary, these results suggested that *ina‐1* mutants further exacerbated the epidermal and muscle junction detachment phenotype by affecting other CeHDs assembly components in *C. elegans* models.

### 2.7 *Ina‐1* Mutation Affects CeHDs Assembly Proteins *Vab‐10a*, *Mup‐4*, and *Let‐805* to Further Disrupt Epidermal‐Muscle Junction Attachment

To provide additional evidence that CeHDs assembly‐related proteins were affected by *ina‐1* mutation and caused structural CeHDs disassociation, multimutations of CeHDs components were utilized to observe variations in epidermal muscle attachment.

**FIGURE 7:**
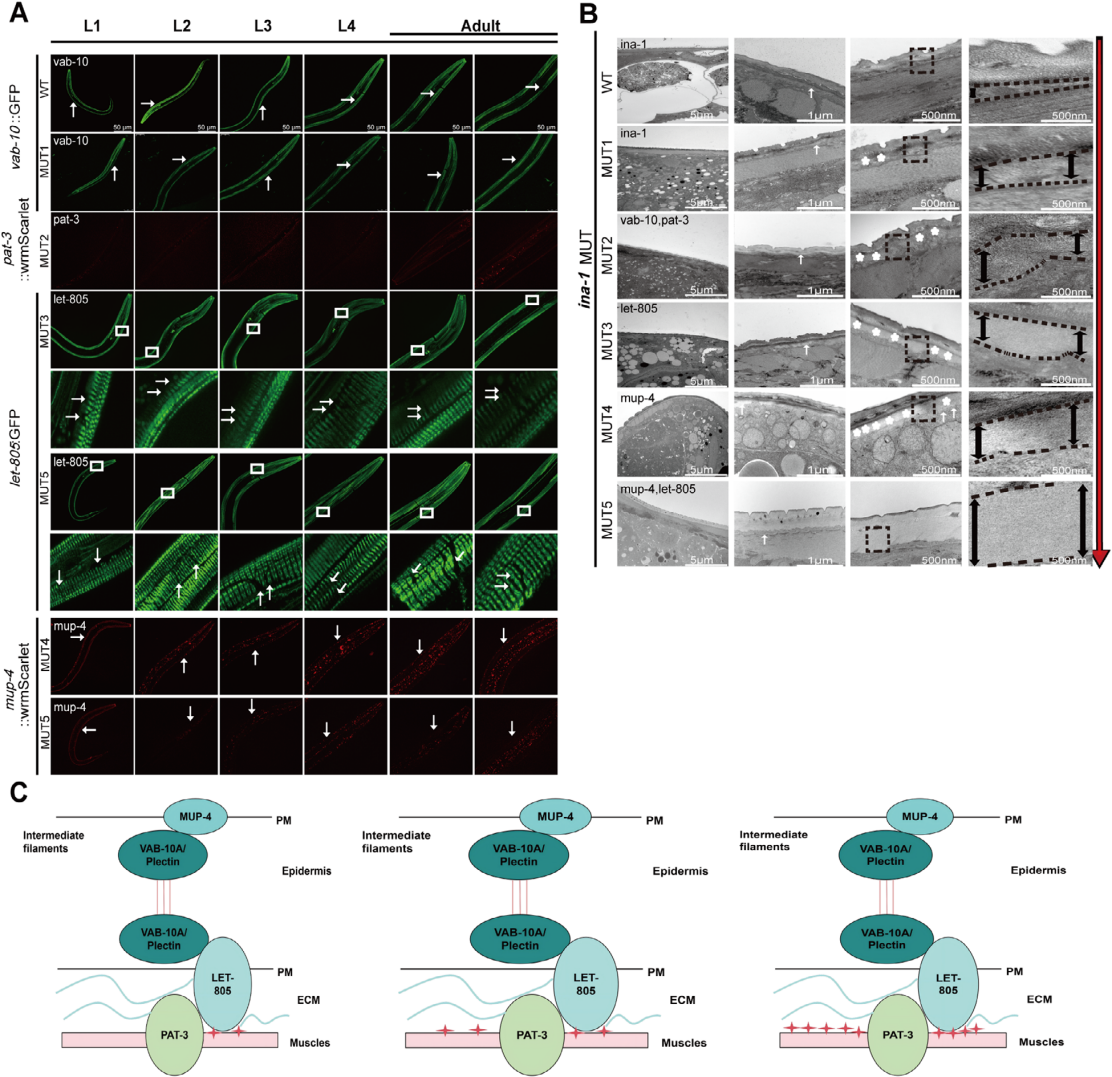
The *ina‐1* mutation affects CeHDs assembly proteins *vab‐10a, mup‐4*, and *let‐805* to further disrupt epidermal muscle junction attachment. (A) Confocal microscopy images show multiple mutants with CeHDs components from L1, L2, L3, and L4 larvae to the adult stage. *ina‐1* mutants with a *C. elegans* background showed reduced fluorescence intensity compared with wild‐type cytolinker *vab‐10a*::GFP localization in MUT1 and control. The *mup‐4* and *let‐805* dense plaques were significantly disrupted in L1–4 larvae and the adult stage. *let‐805*::GFP mutation showed worse dense plaque damage in MUT5 than in the MUT3 type. The *mup‐4*::wrmScarlet mutation showed worse dense plaque damage in MUT5 than in the MUT4 type, losing fluorescence intensity at the epidermis. Arrows show the compared *C. elegans* region. Scale bar: 50 µm. (B) Larger gaps and more separation of epidermal and muscle junctions were consistently present from MUT1 to MUT5 compared with wild type. More asterisks mean larger gaps and more separation. Arrows show the same size of *C. elegans* tissue with a widening gap. Pat 4 represents high magnifications of the region highlighted by the dashed rectangles in multimutants. Scale bar: 5 µm, 1 µm, and 500 nm. (C) Diagram of disruption in epidermal and muscle junction structures, with increasing level of disrupted CeHDs. More asterisks indicate larger gaps and more separation.

Confocal results showed multiple mutants in CeHDs components from L1, L2, L3, and L4 larvae and the adult stage. The *ina‐1* mutants showed reduced fluorescence intensity compared with the wild‐type cytolinker *vab‐10a*::GFP. Dense plaques in apical *mup‐4* (red) and basal *let‐805* (green) CeHDs were significantly disrupted in L1–4 larvae and the adult stage. Basal CeHDs *let‐805* mutation showed enhanced damage in dense plaques in MUT5 compared with those in MUT3, mainly in the epidermis from L1–4 larvae and the adult stage. Furthermore, *mup‐4*::wrmScarlet mutation showed increased damage in dense plaques in MUT5 compared with those in MUT4 from L1–4 larvae and the adult stage, with most MUT5 *C. elegans* presenting with loss of fluorescence at the epidermis (Figure 7A). Larger gaps and more epidermal and muscle junction separation were consistently present across MUT1 to MUT5 compared with wild‐type samples (Figure 7B). A diagram of the epidermal and muscle junction structure disruption showed the more severe phenotype occurring in the context of the increasing level of disrupted CeHDs (Figure 7C).

In summary, the study results further validated that *ina‐1* mutants affected CeHDs assembly protein cytolinker (*vab‐10a*) and apical (*mup‐4)* and basal *(let‐805)* CeHDs to further disrupt epidermal muscle junction attachment. This mimicked the mutations in genes relevant to HD assembly that caused dermal and epidermal separation with blistering characteristics of the PD spectrum (Graphical abstract).

### 2.8 Mutation‐Specific Transcriptomic and Serum Proteomic Signatures of ITGA6 Deficiency

RNA‐seq analysis was performed on two ITGA6 mutant keratinocyte (Ker‐CT) models carrying either a missense or a loss‐of‐function variant. GO enrichment analysis showed that both mutant lines exhibited alterations related to epithelial and epidermal development, consistent with the role of ITGA6 in maintaining epithelial integrity. KEGG pathway analysis revealed distinct mutation‐specific profiles: the missense variant was predominantly enriched in the NOD‐like receptor signaling pathway (Figure S2A–C), whereas the loss‐of‐function variant was enriched in NOD‐like receptor, TNF, and MAPK signaling pathways (Figure S2D–F). Comparative analysis between the missense and loss‐of‐function models further highlighted that the loss‐of‐function variant showed stronger enrichment in TNF, MAPK, and Wnt/β‐catenin signaling pathways (Figure S2G–I), suggesting a more pronounced effect on inflammatory and developmental signaling networks. To complement these transcriptomic findings, Olink proteomic profiling was conducted on patient sera, which demonstrated enrichment in NF‐κB, TNF, and pyroptosis pathways. Consistently, several upregulated genes identified in the volcano plot, including IL‐8, TNFSF14, CCL3, CCL4, MCP‐3, CD40, OSM, LIF, and EN‐RAGE, represent canonical mediators of inflammatory and immune activation. In addition, regulatory molecules such as SIRT2, AXIN1, ST1A1, STAMBP, and ADA were also elevated, suggesting secondary modulatory effects or crosstalk with related pathways (Figure S3A–D). These findings highlight the central role of NF‐κB/TNF‐driven signaling in the observed transcriptional landscape.

## 3 Discussion

The critical role of HD‐related genes is apparent in inherited severe blistering disorders, providing a clear schematic of how pathogenic mutations disrupt the HD function [2, 3, 5, 14, 15, 35, 36]. In the present study, 58.9% of PD patients carried at least one pathogenic variants in one of 16 HD‐related genes. Among them, *ITGA6* was identified as the candidate gene and validated in PD patients, with acquired autoimmune bullous disorders including BP, EBA, DH, and LigA subtypes. The *ITGA6* is an essential HD component and a hub of the HD protein‐interaction network [5, 8]. A previous study suggested that there may be interactions among HD‐related genes, especially when one of the genes is damaged or abnormally expressed [8]. In addition, structural damage in the *C. elegans* epidermis induced an innate immune response [37]. In the present study, the associated experiments in *C. elegans* indicated that *ina‐1*, a homologue of the *ITGA6* gene, may play an important role in PD pathogenesis, affecting cytolinker (*vab‐10a*) [38] and apical (*mup‐4)* [39] and basal (*let‐805*) CeHDs [40] assembly proteins to further disrupt epidermal muscle junction attachment.

Despite the well‐documented effect of pathogenic genetic variants on specific protein complexes in congenital EB [41, 42, 43], the role of pathogenic mutations in the acquired blistering diseases is largely unknown. For example, loss‐of‐function germline mutations in *Dsg1* result in impaired desmosome formation in the upper epidermal layers, abnormal differentiation, and acantholysis [44]. A *BP180* dysfunction mouse model developed severe spontaneous skin inflammation characterized by immune cell infiltration, increased IgE concentration, pruritus, and defective skin barrier [35]. Lack of collagen XVII expression resulted in weakened dermal–epidermal adhesion and autoimmune bullous disease along the basement membrane [45, 46]. Thus, it was hypothesized that rare pathogenic variants in genes encoding HD assembly proteins may lead to the occurrence of autoantibodies, leading to the true dermo‐epidermal separation and subepidermal blister formation.

Loss of *PLEC* and *BP230* affected HD formation and induced mechanical fragility of the skin, but to a lesser extent than integrin loss, because plaques were still observed. Keratins failed to attach to defective HDs in *BP230* knockout (KO) mice, whereas they were absent only in areas undergoing blistering and histolysis in *PLEC* KO mice [47]. The assembly of mature HD was dependent on the recruitment of laterally localized *ITGA6* at a later stage. An insufficient amount of *ITGA6* protein resulted in nonfunctional HDs in zebrafish models [47]. In vitro, cleaved laminin‐332, the high‐affinity ligand for a6b4‐integrin, stabilized HDs, while unprocessed laminin‐332 triggered their disassembly and turnover [48]. Recently, it has been shown that Wnt/β‐catenin signaling regulates the localization of HD components in keratinocytes and that the atypical protein kinase C pathway was involved in Wnt inhibition‐induced HD disarrangement in *PLEC* KO cells or cells with PLEC‐type XVII collagen binding defects [49]. BP230 incorporation into HD structures required a previous interaction between *PLEC* and a6b4‐integrin and *BP180* recruitment, which was depicted in a hierarchical model for the assembly of HD structures [7, 50]. This evidence suggests that damage to assembly component proteins affects HD structure stability. The absence or decreased expression of key proteins in the HD can have a great impact on its formation, as demonstrated in recent studies using both vertebrate and invertebrate model systems [51]. For example, the phosphorylation state of the *ITGB4* cytoplasmic domain determined the strength of its interaction with *PLEC* and may act as a regulatory point for HD assembly and disassembly [51]. Although our previous experiments indicated that *ITGA6* (extracellular matrix receptor) and *PLEC* played a central role in modulating HD disassembly, the mechanisms changing HD‐related genes in vivo after acquired destruction remained poorly understood. *C. elegans* provides a powerful genetic model to address this issue [52]. A novel *C. elegans* model was used to validate the hypothesis in the present study.

Analysis of the clinical data (Table S3) revealed that PD patients carrying mutations in hemidesmosome‐related genes exhibited variable disease severity and clinical outcomes. Notably, variants in genes such as ITGA6 and LAMC2 were more frequently observed in patients with severe mucocutaneous involvement and a refractory disease course. These findings suggest that HD gene mutations may not only predispose to PD development but also modulate the clinical phenotype and prognosis. This observation is consistent with previous reports linking structural defects in epithelial adhesion complexes to increased tissue fragility [53]. Although our current study cannot fully delineate the mechanistic basis of these associations, the integration of genetic and clinical data provides a starting point for stratifying patients according to their genetic background, which may ultimately guide personalized therapeutic strategies. In addition, the reduced protein expression observed in heterozygous carriers is most consistent with haploinsufficiency, although potential epistatic interactions with specific HLA alleles may further influence immune recognition and contribute to clinical heterogeneity. CeHDs in *C. elegans* model animals were structurally, molecularly, and functionally comparable to vertebrate HDs in skin [37, 54]. In human skin, HD‐like plaques first appear at about nine weeks of gestational age, and their number increases over the next six weeks [37, 55]. The association between HD structures and intermediate filaments also takes place during this period. HD structures appear fully developed by 15 weeks of gestation [55]. This is why all types of *C. elegans* from L1–4 to adult stages were observed in order to validate changes in the relevant CeHDs components. Interestingly, the study results also confirmed that *ina‐1* mutations disrupted the structure of CeHDs in *C. elegans* from L1–4 to adult stages. The extent of this effect was related to the degree of *ina‐1* mutation impact affecting other structurally relevant CeHDs components, such as cytolinker (*vab‐10a*) and apical (*mup‐4*) and basal (*let‐805*) CeHDs proteins.

Based on these results, the study further verified at the phenotypic and protein expression levels that the *ITGA6* (*ina‐1*) variant in model animals may disrupt the assembled HD structure components, thereby affecting normal skin junctions and facilitating PD development. These findings pointed to a novel role of HD assembly gene mutation in the development of not only inherited but also acquired skin‐blistering disorders. *C. elegans* models the structural consequences of hemidesmosome disruption, not the autoimmune process itself. The study also shed light on the genetic pathogenesis of acquired autoimmune bullous disorders in addition to previously reported HLA associations.

Our multi‐omics analyses reveal both shared and mutation‐specific consequences of ITGA6 disruption. Transcriptomic profiling showed that missense variants and loss‐of‐function variants both preferentially activated the NOD‐like receptor pathway, whereas loss‐of‐function variants also affected MAPK and TNF signaling, in addition to common enrichment in epithelial and epidermal development. Furthermore, in the comparative analysis between the missense and loss‐of‐function models, we identified enrichment of the Wnt/β‐catenin signaling pathway, which is consistent with previous reports [49]. These data indicate that HD dysfunction can influence keratinocyte biology through distinct immunoinflammatory and developmental axes depending on the mutational context. Proteomic profiling of patient sera provided convergent evidence, with enrichment of NF‐κB, TNF, and pyroptosis pathways. The alignment between transcriptomic and proteomic findings suggests that missense mutations may drive systemic inflammation through NOD‐like receptor–NF‐κB–TNF signaling and pyroptotic responses, while loss‐of‐function variants perturb broader developmental and inflammatory networks. These findings suggest that the observed protein expression changes are not solely due to haploinsufficiency but may involve broader immune regulatory dysfunction, potentially including epistatic interactions with MHC‐related pathways (Figures S2, S3). This discussion now serves as a conceptual framework to guide subsequent investigations into hemidesmosome regulatory networks. Importantly, the identification of TNF signaling highlights actionable pathways, as JAK inhibitors have demonstrated efficacy in PD [56, 57, 58, 59, 60, 61]. These findings not only elucidate mechanistic links between ITGA6 mutations and downstream signaling cascades but also provide a rationale for evaluating targeted interventions in clinically defined patient cohorts.

There are some limitations in our study. Recent studies have reported COL7A1 variants in patients with EBA, providing preliminary support for their role in disease pathogenesis [12, 13]. Despite these findings, COL7A1 was not included in our initial gene panel, a limitation we address in the current analysis. In addition, as shown in Table S1, the age distribution varied across subtypes, which is consistent with their known epidemiology: BP usually occurs in the late 70s [62], LigA shows peaks before 5 years and after 60 years [63], DH has a mean onset age around 50 years [21], and EBA presents with a median onset age of 44 years [64]. Such heterogeneity may influence clinical manifestations and limit generalizability, although our analyses focused on shared molecular features to minimize age‐related bias. Finally, we were unable to further delve into the downstream mechanisms, such as how matrix metalloproteinases (MMPs) and granzyme B, among others, further promote the disruption of the dermal–epidermal junction in patients carrying susceptibility genes. Although previous studies have demonstrated that MMPs and granzyme B are highly expressed at the dermal–epidermal junction in patients with PD, integrins (ITGA6, ITGB4), collagen VII, and collagen XVII are reduced or absent in the blistering areas (Figure S4). Additionally, previous studies have shown that MMP‐9 is essential for subepidermal blistering in experimental BP and is capable of degrading recombinant BP180 and other extracellular matrix components, which represents a critical step leading to dermal–epidermal separation. Furthermore, the activation of MMP‐9 (from its inactive proMMP‐9 form to its active actMMP‐9 form) in the early stages of BP primarily relies on tissue plasminogen activator (tPA). The plasminogen/plasmin system may contribute to neutrophil recruitment by activating MMP‐9 [65, 66, 67, 68, 69, 70, 71, 72, 73, 74].

The choice of C. elegans was further supported by its established utility in modeling extracellular matrix (ECM)‐anchoring junctions. For instance, mutations in ITGA6 cause detachment of the hypodermis from the basement membrane, phenocopying human epidermolysis bullosa [10]. Additionally, *C. elegans* permits live imaging of hemidesmosome‐like structures via fluorescent reporters (e.g., vab‐10a::GFP), enabling dynamic assessment of structural disruptions. While murine models offer closer physiological relevance, their complexity limits large‐scale genetic interaction studies. Our approach bridges this gap by combining *C. elegans* genetics with human clinical data, a strategy validated in prior studies of collagen IV and laminin mutations. Our findings of pathogenic variants in hemidesmosome‐related genes suggest potential overlaps with autoimmune blistering diseases such as BP. Although the exact mechanisms linking hemidesmosome mutations to BP pathogenesis remain unclear, our data provide a rationale for further investigation into how these genetic variants might contribute to the development or modulation of autoimmune blistering disorders.

The present study reported on the association of HD assembly‐related gene variants with the development of not only inherited but also acquired autoimmune bullous disorders. A novel *C. elegans* model was established to explore the functional consequences of genetic variation in CeHD structure, mimicking epidermal dermatosis junction separation observed in PD patients. In addition, the study findings pointed to a novel role of *ITGA6* variants and their effect on other HD assembly‐related genes disrupting HD structure, such as *vab‐10a*, *mup‐4*, and *let‐805*.

## 4 Materials and Methods

### 4.1 Patient Characteristics

The present study was approved by the ethics committees at the Shandong Provincial Institute of Dermatology and Venereology. Written informed consent was obtained from all participants or their legal guardians prior to enrollment. The patients were enrolled in the study if they presented with pruritic erythema and tense blisters and met the following immune test criteria. Immune test criteria of pemphigoid disease (PD): (1) BP: linear C3/IgG deposition in the BMZ region and BP180‐ and/or 230 serum‐positive; (2) EBA: linear C3/IgG deposition in the BMZ region and indirect immunofluorescence (IIF) (salt‐split human) deposition in the dermis or COL7 serum‐positive; (3) LigA: linear IgA deposition in the BMZ region; and 4) DH: granular or fibrillar IgA deposition in the papillary tips or anti‐endomysial antibody (EMA)‐ or anti‐tTG‐positive. PD diagnosis relied on typical clinical and histological presentations, alongside direct or indirect immunofluorescence examinations. PD samples without HD‐related gene mutation were obtained as controls.

The total score of disease association (disease severity and outcome) was defined by three indicators, including IIF of IgG titers, cumulative duration of medication administration (months), and disease outcome (complete remission off therapy, maintenance therapy, and mortality). The IIF score was defined according to the principle of increasing IgG antibody dilution, where 0 indicates negative, 1 corresponds to a titer of 1:10, and 8 corresponds to 1:1280. Complete remission off therapy was defined as the absence of new or established lesions or pruritus while the patient was off all PD therapy for at least 2 months.

Furthermore, patients with prednisone equivalent (PE) ≥100 mg were defined as “very severe”, those with PE of 80–90 mg were “severe”, and individuals with PE ≤60 mg were defined as “mild to moderate”.

### 4.2 Genotyping/Data Analysis

An HD assembly 16‐gene targeted capture panel was designed to cover the entire gene sequence, including 10 kb upstream and downstream, and all coding exons and introns. Genomic capture from the pooled libraries was carried out using the TargetSeq Enrichment Kit (iGeneTech) as described previously. The libraries were sequenced on an Illumina HiSeq 3000 platform in lanes and generated 2 × 150–base pair (bp) paired‐end reads. The sequencing output was deconvoluted into individual sample reads and sorted using Picard tools. Reads were aligned to the hg19 reference sequence using bwa. Duplicate reads were identified and marked using Picard tools. Alignments were further refined for localized realignment around indel sites, and recalibration of the quality scores was performed using GATK tools.

Variants were excluded if the allele frequency was >0.005 in the GnomAD all and Asian database or the 1000 Genomes project all and Asian. All of the variants were then annotated and assigned to one or more genes using the Annovar annotation pipeline. Variants with multiple functional annotations were classified according to the annotation predicted to be the most damaging within each assigned gene. Pathogenic variants were characterized as splice acceptor, splice donor, stop gained, frameshift, stop lost, start lost, or transcript amplification and included the categories of in‐frame insertion, in‐frame deletion, missense variant, or protein alteration. Missense variants were considered predicted as damaged or probably damaged using SIFT, Polyphen2, or Condel software.

### 4.3 Immunohistochemistry

Skin tissue samples with both perilesional (adjacent to lesions) and nonlesional (clinically normal) areas of BP patients were fixed using 4% paraformaldehyde before embedding in paraffin. The prepared tissue sections (4 µm) were then baked for 2 h at 65°C, followed by dewaxing with xylene and rehydration with graded alcohol. After rehydration, the sections were stained using IHC kits (#NEL820001KT, AKOYA Biosciences) following the manufacturer's instructions. Scanning images of tissue samples were obtained using the Akoya Phenocycler Fusion (AKOYA Biosciences). Image processing was carried out using Phenochart (version 1.2.0) and Inform (version 2.6.0) software. Antibodies for IHC experiments, including *LAMC2* (#ab210959, Abcam), *EPPK1* (#ab247172, Abcam), *ITGA6* (#ab181551, Abcam), *LAMB3* (#ab97765, Abcam), *JUP* (#ab2309, Abcam), *ITGB4* (#ab14803, CST), *LAMA3* (#ab242197, Abcam), DST (#ab244440, Abcam), and *PLEC* (#ab32528, Abcam), were used according to the recommended dilution and incubation times. Quantitative analysis was performed using ImageJ software.

### 4.4 *C*. elegans Models

Based on *ina‐1* mutant nematodes with a *C. elegans* background, a variety of mutant *C. elegans* models were constructed to investigate changes in CeHDs components. Control *ina‐1* N2 and other strains were grown on Nematode Growth Medium (NGM) plates seeded with OP50 *Escherichia coli* at 20°C unless otherwise stated (Brenner, 1974). The following alleles were used: *vab‐10a, pat‐3, let‐805*, and *mup‐4*, which were outcrossed three or four times against N2 *ina‐1* and maintained in trans to the balancer qC1 [dpy‐19(e1259) glp‐1(q339)]. First, wild‐type *ina‐1 C. elegans* was constructed using CRISPR‐Cas technology to serve as the control. In addition, mutant types MUT1, MUT2, MUT3, MUT4, and MUT5 were generated. MUT1 was double‐mutant with *vab‐10a::GFP I; ina‐1::wrmScarlet III* (corresponding to the human *ITGA6*(Asp230Glufs Ter6)), MUT2 was double‐mutant with *vab‐10a*::GFP; *pat‐3*::wrmScarlet; *ina‐1::wrmScarlet III*, MUT3 was defined as *vab‐10a*::GFP; *let‐805*::wrmScarlet; *ina‐1::wrmScarlet III*, MUT4 was defined as triple‐mutant with *vab‐10a*::GFP; *mup‐4*::wrmScarlet; *ina‐1::wrmScarlet III* and MUT5 was defined as tetra‐mutant with *let‐805*::GFP; *mup‐4*::wrmScarlet; *ina‐1::wrmScarlet III*. Wild‐type and *lam‐2* (*LAMC2*(−10 bp)) *C. elegans* mutants were simultaneously constructed to explore the effect of the *LAMC2* gene on HD structure.

### 4.5 Transmission Electron Microscopy

Perilesional and nonlesional skin biopsies from BP patients were fixed in 3% glutaraldehyde in 0.1 M cacodylate buffer (pH 7.4) at room temperature, cut into ∼1 mm^3^ pieces, washed, postfixed with 1% osmium tetroxide at 4°C for 1 h, rinsed, dehydrated through graded ethanol, transferred to propylene oxide, and embedded in epoxy resin. Sections were analyzed using a TEM (JEOL‐1200, Weiya Bio Co., Ltd). HDs and intercellular desmosomes of basal keratinocytes were imaged and evaluated in PD patients, including those with BP, LigA, and DH. HD profiles were defined, and parameters were assessed across PD subtypes. Images at 30,000× magnification were used for quantification, with >50 desmosomes/HDs evaluated per condition per experiment. In C. elegans models, TEM micrographs of adult wild‐type and ina‐1 mutant CeHD structures—including hypodermis, basal cuticle, and adjacent body wall muscle—were compared.

### 4.6 Confocal Microscopy

Images of wild‐type *C. elegans* and multimutant (MUT1, MUT2, MUT3, MUT4, and MUT5) *ina‐1* and wild type *Iam‐2* with the developmental process from L1–L4 larvae to adult were obtained using confocal microscopes (Leica TCS SP8). Quantitative analysis of *C. elegans* L1–L4 larvae and adults used the relative intensity data and was performed in ImageJ (five cases for each group).

### 4.7 Serum Protein Profiling by Proximity Extension Assay

Serum samples collected from study participants were preserved in liquid nitrogen until analysis. Protein profiling was performed using the proximity extension assay (PEA) combined with quantitative PCR, implemented on the Olink Proseek Multiplex ultrasensitive platform with the Target 96 Organ Damage panel (Olink Proteomics, Uppsala, Sweden). Normalized protein expression (NPX) values were generated in accordance with the manufacturer's protocol.

### 4.8 Cell Culture and RNA Extraction, Library Preparation, and Sequencing

The immortalized human keratinocyte line Ker‐CT (ATCC CRL‐4048; Lot No. 70004879) was obtained from ATCC and maintained in KGM‐Gold Complete Medium (Lonza, Cat. No. 00192060) under standard culture conditions (37°C, 5% CO_2_, humidified incubator). For RNA sequencing, 1 × 10^6^ cells were seeded into 12‐well plates and cultured for 24 h. Stable Ker‐CT cell lines overexpressing wild‐type (WT) ITGA6 or ITGA6 variants were generated by lentivirus‐mediated transduction and selected with hygromycin (50 µg/ml; HY‐K1051, MCE). Control lines were created using empty vectors. Two ITGA6 mutant Ker‐CT cell lines were generated via gene synthesis. For the missense variant, the NM_000210 transcript was used as the template, and the A699C point mutation was introduced according to the experimental design. For the loss‐of‐function variant, the NM_001316306 transcript was used, and the 341dupA mutation was incorporated. In both cases, the corresponding full‐length cDNA sequences carrying the desired mutations were synthesized by a commercial provider and subsequently cloned into appropriate expression vectors for stable transfection into Ker‐CT cells. Successfully transfected cells were selected and expanded for downstream transcriptomic and functional analyses. All cell lines used in this study were authenticated by short tandem repeat (STR) profiling and confirmed to be free of mycoplasma contamination before the commencement of experimental work.

Total RNA was extracted with the RNAiso Plus kit (Takara, Japan). RNA integrity was verified by gel electrophoresis, and concentration was determined with a Qubit fluorometer (Thermo Fisher Scientific, Waltham, MA, USA). mRNA was enriched using the NEBNext Poly(A) mRNA Magnetic Isolation Module, and libraries were constructed with the NEBNext Ultra II RNA Library Prep Kit (Illumina), following the manufacturer's protocols. Library quality was assessed by concentration measurement with the Qubit dsDNA HS Assay Kit (Thermo Scientific), fragment distribution with the Agilent 4200 system, and molar concentration with the KAPA Library Quant Kit (Illumina).

Sequencing was carried out on the Illumina NovaSeq 6000 platform by Genergy Biotechnology Co., Ltd. (Shanghai, China), with technical support from Shanghai iProteome Biotechnology Co., Ltd. Raw sequencing data were processed using Skewer, quality‐checked by FastQC (v0.11.2), and mapped to the human reference genome (hg38) using STAR, with transcript assembly performed by StringTie.

### 4.9 Statistics and Reproducibility

Data are representative of at least three biological replicates with similar results. Details of statistical analyses are reported in the Figure legends. Statistical analyses were performed using GraphPad Prism, and Adobe Illustrator was used to make the figures.

## Author Contributions

Y.H.S. and F.R.Z. designed this study. Data curation and formal Analysis: Y.H.S., S.C. and Z.Z.W.; Investigation and performed experiments: S.C., T.Y.W., C.L., S.S.M., G.Q.Y., Q.Q.X., T.T.L., Y.Q.Y., L.L.S., Q.Z., C.W., Y.X.L., S.L.C., J.W.W., and G.Z.Z.; Visualization: S.C., Y.H.S, and X.Y.P.; Writing – original draft: S.C., and Y.H.S.; Writing – review & editing: S.C., Y.H.S., H.L., and F.R.Z. All authors have read and approved the final manuscript.

## Funding

This work was supported by the Taishan Scholars Program of Shandong Province (tsqn201909141), the Outstanding Youth Grant of Shandong Natural Science Foundation (ZR2020YQ56), and the National Natural Science Foundation of China (82573983 & 82073441).

## Conflicts of Interest

The authors declare no conflicts of interest. The authors declare that the capture probes used in this study were custom‐designed and synthesized by iGeneTech (Beijing, China), and the detailed probe sequences are proprietary. The authors have no financial or personal relationship with the company.

## Ethics Statement

This study was approved by the institutional review board (IRB) committees at the Shandong Provincial Institute of Dermatology and Venereology, Shandong Academy of Medical Science (20220310KYKTKS010). All participants provided written informed consent. Animal experiments were approved by the Institutional Animal Care and Use Committee of Shandong Provincial Institute of Dermatology and Venereology, Shandong Academy of Medical Science (W202203070192).

## Supporting information




**Supporting Information file 1**: mco270627‐sup‐0001‐SuppMat.docx

## Data Availability

The data underlying this article are available from the corresponding author upon reasonable request.
