# Supplementary material for: Hemidesmosome Mutations Contribute to the Onset and Severity of Acquired Autoimmune Bullous Diseases
Source: MedComm (2020). 2026 Feb 15;7(3):e70627. doi: 10.1002/mco2.70627 (PMC12906664; doi:10.1002/mco2.70627)
Supplement: Supplementary file 1 — Supporting Information file 1: mco270627‐sup‐0001‐SuppMat.docx [file MCO2-7-e70627-s001.docx]

**Supplementary Information**

**Hemidesmosome Mutations Contribute to the Onset and Severity of Acquired Autoimmune Bullous Diseases**

**Running head: Hemidesmosome mutations and pemphigoid diseases**

**Authors:** Shan Cao^1,2^, Tianyu Wang^1,2^, Chen Lv^1,2^, Shanshan Ma^1,2^, Gongqi Yu^1,2^, Qianqian Xia^1,2^, Tingting Liu^1,2^, Yueqian Yu^1,2^, Lele Sun^1,2^, Xiaoyan Pei^1,2^, Qing Zhao^1,2^, Zhenzhen Wang^1,2^, Chuan Wang^1,2^, Yongxia Liu^1,2^, Shengli Chen^1,2^, Jianwen Wang^1,2^, Guizhi Zhou^1,2^, Hong Liu^1,2^, Yonghu Sun^1,2^*, Furen Zhang^1,2^*

**Affiliations:**

1. Dermatology Hospital of Shandong First Medical University, Jinan 250022, Shandong, China;

2.Shandong Provincial Institute of Dermatology and Venereology, Shandong Academy of Medical Sciences, Jinan 250022, Shandong, China.

***Corresponding authors**: Yonghu Sun (sunyh@sdfmu.edu.cn) and Furen Zhang (frzhang@sdfmu.edu.cn).

Addresses: Dermatology Hospital of Shandong First Medical University; Shandong Provincial Institute of Dermatology and Venereology, Shandong Academy of Medical Sciences. 27397 Jingshi Lu, Jinan 250022, Shandong Province, P.R. China

**All author’s email addresses:**

Shan Cao: [shancaolily@163.com](mailto:shancaolily@163.com); Tianyu Wang: [WTyu0418@163.com](mailto:WTyu0418@163.com); Chen Lv: [312642632@qq.com](mailto:312642632@qq.com); Shanshan Ma: [mashanshan5000@163.com](mailto:mashanshan5000@163.com); Gongqi Yu: [yugongitje@163.com](mailto:yugongitje@163.com); Qianqian Xia: [119419273@qq.com](mailto:119419273@qq.com), Tingting Liu: [qianqianltt@126.com](mailto:qianqianltt@126.com); Yueqian Yu: [yueqian.yu@outlook.com](mailto:yueqian.yu@outlook.com); Lele Sun: [438065194@qq.com](mailto:438065194@qq.com); Xiaoyan Pei: [2568364042@qq.com](mailto:2568364042@qq.com); Qing Zhao: [zhongguowawaqing@163.com](mailto:zhongguowawaqing@163.com); Zhenzhen Wang: [zzwang1030@163.com](mailto:zzwang1030@163.com); Chuan Wang: [wangchuan86@126.com](mailto:wangchuan86@126.com); Yongxia Liu: [liuyx_comeon@163.com](mailto:liuyx_comeon@163.com); Shengli Chen: [shengli28@163.com](mailto:shengli28@163.com); Jianwen Wang: [470894013@qq.com](mailto:470894013@qq.com); Guizhi Zhou: [zhou_guizhi2003@163.com](mailto:zhou_guizhi2003@163.com); Hong Liu: [hongyue2519@hotmail.com](mailto:hongyue2519@hotmail.com); Yonghu Sun: sunyh@sdfmu.edu.cn; Furen Zhang: [frzhang@sdfmu.edu.cn](mailto:frzhang@sdfmu.edu.cn).

[Supplementary Information 1](#_Toc30827)

[Figures 3](#_Toc2243)

[Figure S1. Pathogenic variants fulfilling the pathogenicity criteria were identified within 15 genes. 3](#_Toc12703)

[Figure S2. ITGA6 mutation inhibits cell development, differentiation, and proliferation. 4](#_Toc15551)

[Figure S3. Targeted Serum Proteome with GO and KEGG Analyses of Differentially Expressed Proteins Indicates Predominant Immune and Inflammatory Signaling. 4](#_Toc18793)

[Figure S4. Upregulation of Protease Markers in Bullous Pemphigoid (BP) Skin Tissues. 5](#_Toc28090)

[Tables 7](#_Toc25733)

[Table S1. Patient demographic, histology and serotype characteristics. 7](#_Toc19678)

[Table S2. Clinical information of subtype with 18 patients of EBA. 8](#_Toc1497)

[Table S3. Clinical information of disease severity and outcome associated with HD assembly 15 hemidesmosome assembly related genes mutation with 201 PD patients. 9](#_Toc15426)

**Figures**

**Figure S1. Pathogenic variants fulfilling the pathogenicity criteria were identified within 15 genes.**

**
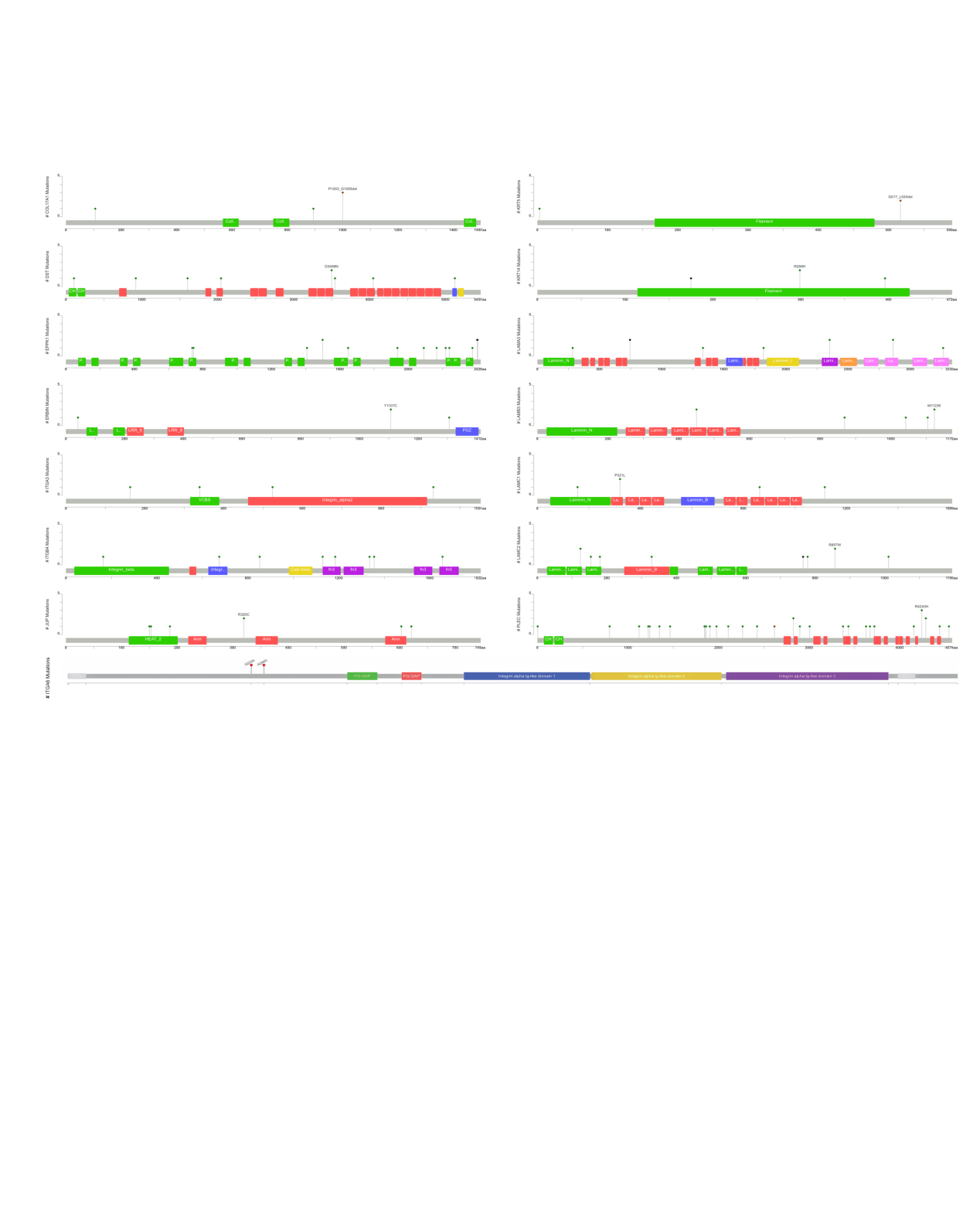
**

**Figure S2. ITGA6 mutation inhibits cell development, differentiation, and proliferation.**

**
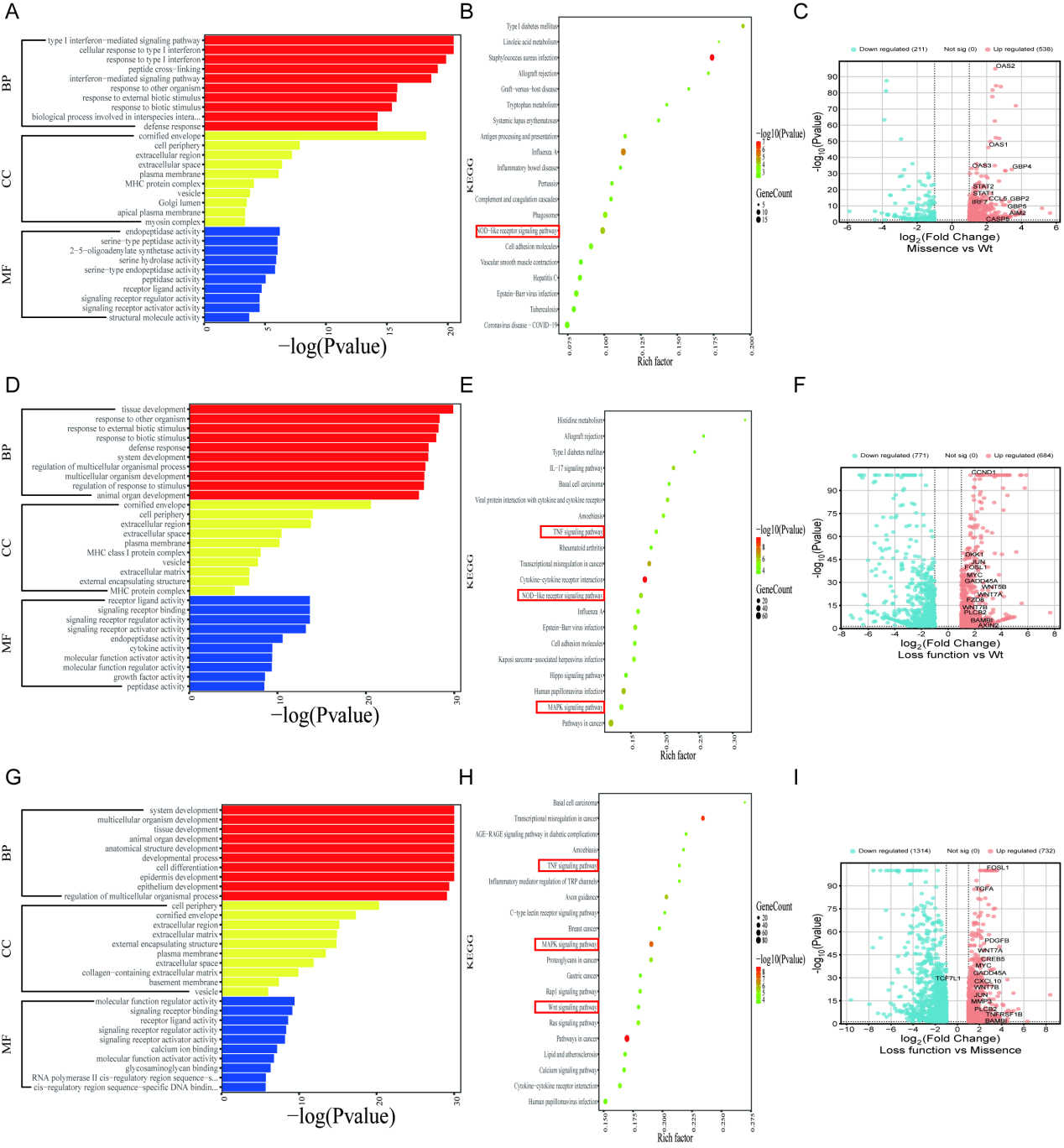
**

(A)GO enrichment through RNA sequencing of Ker-CT cells with missence ITGA6 overexpression. (B) KEGG pathway analysis highlighting the NOD-like receptor signaling pathway. (C) Volcano plot showing differentially expressed genes associated with NOD-like signaling in the ITGA6 missense variant model (log2FC≥1, P≤ 0.05). (D) GO enrichment through RNA sequencing of Ker-CT cells with loss-of-function ITGA6 overexpression. (E) KEGG pathway analysis highlighting the NOD-like receptor, MAPK, and TNF signaling pathways. (F) Volcano plot showing differentially expressed genes associated with NOD-like signaling in the ITGA6 loss-of-function variant model (log2FC≥1, P≤ 0.05). (G–I) Comparative analysis between the missense and loss-of-function models: GO enrichment (G) KEGG pathway analysis highlighting TNF, MAPK, and Wnt/β signaling pathways. (H) and volcano plot illustrating representative differentially expressed genes within these pathways (I).

**Figure S3. Targeted Serum Proteome with GO and KEGG Analyses of Differentially Expressed Proteins Indicates Predominant Immune and Inflammatory Signaling.**

**
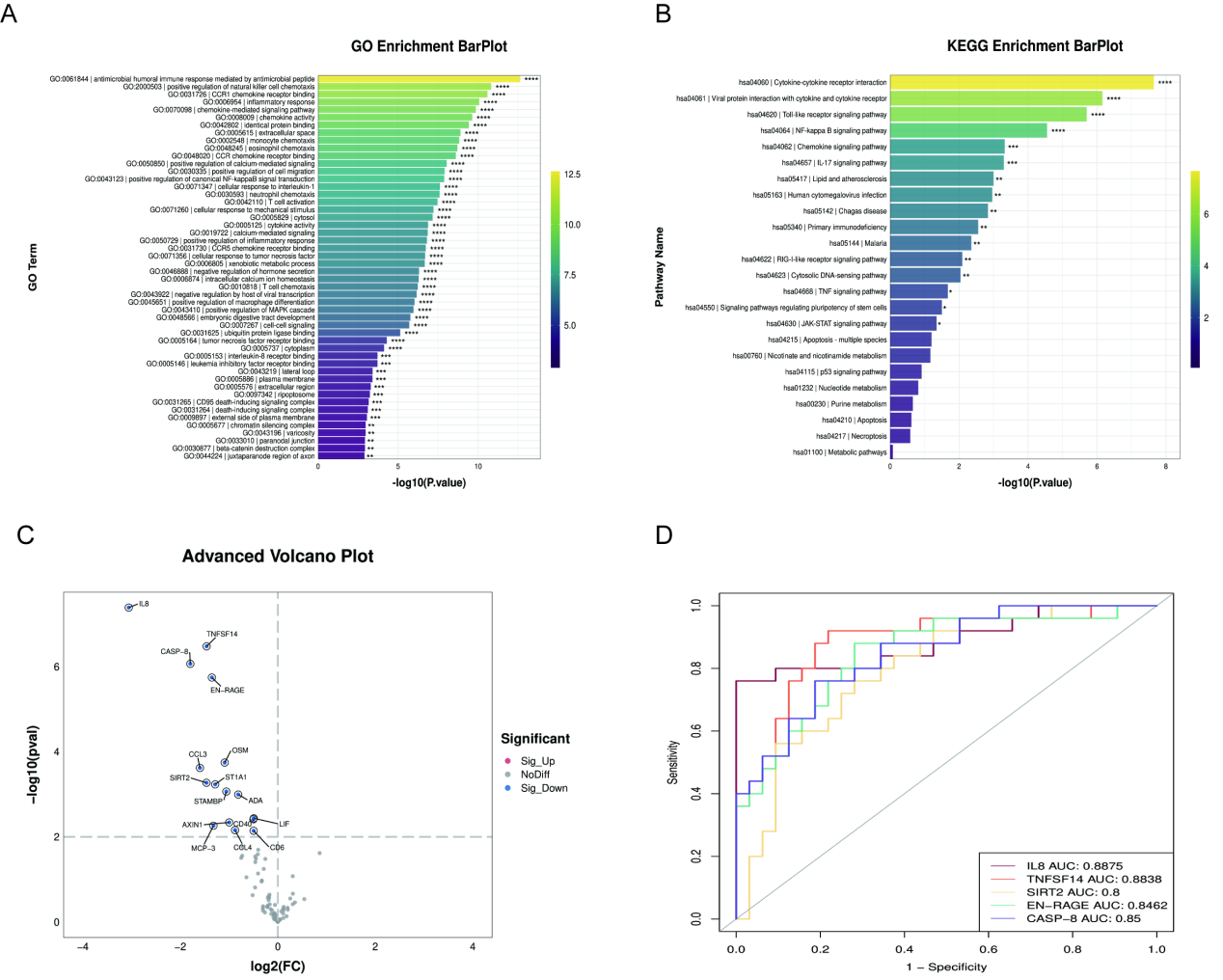
**

(A, B) Elevated proteins in PD patients were defined as the top 50 proteins with the most significant P-values (P≤0.05), based on the screening dataset interrogating 96 serum proteins. GO and KEGG pathway analysis highlighting the NF-kB、TNF signaling pathways. (C) Volcano plot showing differentially expressed genes associated with NF-kB、TNF signaling pathways. (D) Sensitity and prediction of PD with IL8, TNFBF14, SIRT2, EN-RAGE, CASP-B proteins. Welch's t test. FC: fold change.

**Figure S4. Upregulation of Protease Markers in Bullous Pemphigoid (BP) Skin Tissues.**


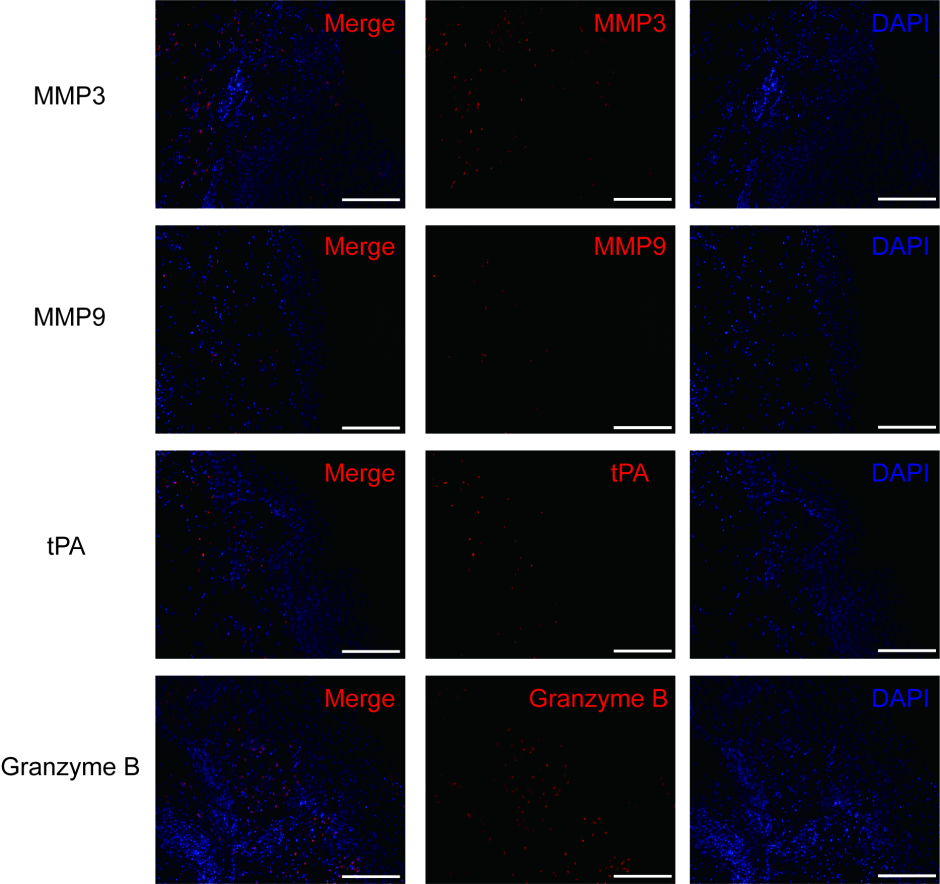


**Tables**

**Table S1. Patient demographic, histology and serotype characteristics.**

| Category | Patients | | | |
| --- | --- | --- | --- | --- |
| Subtype | BP | EBA | LigA | DH |
| Gender |  |  |  |  |
| Male | 53 | 9 | 22 | 26 |
| Female | 40 | 9 | 29 | 14 |
| Total | 93 | 18 | 51 | 40 |
| Age |  |  |  |  |
| Mean | 63 | 51 | 42 | 47 |
| Range | 23~91 | 8~72 | 1~86 | 12~73 |
| Histology |  |  |  |  |
| DIF microscopy performed No. (%) | 93 (100%) | 18 (100%) | 51 (100%) | 40 (100%) |
| Linear |  |  |  |  |
| C3 | 93 (100%) | 18 (100%) | 4 (7.8%) | 2 (5%) |
| IgG | 71 (76.3%) | 16 (88.8%) | 2 (3.9%) | 0 |
| IgA | 64 (68.8%) | 11 (61.1%) | 51 (100%) | 2 (5%) |
| Granular IgA | 0 | 0 | 0 | 36 (90%) |
| Fibrillar IgA | 0 | 0 | 0 | 4 (10%) |
| Serotype |  |  |  |  |
| Serotype positive performed No.(%) | 93 (100%) | 18 (100%) | 23 (54.8%) | 19 (47.5%) |
| BP180 | 93 (100%) | 0 | 0 | 0 |
| BP230 | 49 (52.7%) | 0 | 0 | 0 |
| COL17 | 0 | 15 (83.3%) | 0 | 0 |
| EMA | 0 | 0 | 0 | 9 (47.4%) |
| IIF |  |  |  |  |
| IIF(salt-split humans) performed No.(%) | 51 (54.8%) | 16 (88.8%) | 0 | 0 |
| Epidermal Deposition |  |  |  |  |
| Positive | 43 (84.3%) | 0 | 0 | 0 |
| Dermal Deposition |  |  |  |  |
| Positive | 1 (1.96%) | 13 (81.3%) | 0 | 0 |

**Table S2.** Clinical information of subtype with 18 patients of EBA.

| **Number** | **Patient** | **Sex** | **Age (years)** | **Subtype** |
| --- | --- | --- | --- | --- |
| 1 | EBA | Female | 48 | Mechanobullous |
| 2 | EBA | Male | 66 | Inflammatory |
| 3 | EBA | Male | 55 | Mechanobullous |
| 4 | EBA | Female | 48 | Mechanobullous |
| 5 | EBA | Male | 72 | Inflammatory |
| 6 | EBA | Male | 64 | Mechanobullous |
| 7 | EBA | Male | 67 | Mechanobullous |
| 8 | EBA | Male | 72 | Inflammatory |
| 9 | EBA | Male | 31 | Mechanobullous |
| 10 | EBA | FeMale | 48 | Mechanobullous |
| 11 | EBA | Female | 25 | Inflammatory |
| 12 | EBA | Male | 63 | Mechanobullous |
| 13 | EBA | Female | 69 | Mechanobullous |
| 14 | EBA | Female | 8 | Mechanobullous |
| 15 | EBA | Female | 60 | Mechanobullous |
| 16 | EBA | Female | 70 | Inflammatory |
| 17 | EBA | Female | 17 | Mechanobullous |
| 18 | EBA | Female | 18 | Mechanobullous |

**Table S3.** Clinical information of disease severity and outcome associated with HD assembly 15 hemidesmosome assembly related genes mutation with 201 PD patients.

| ht_ | Mutation gene | Diagnosis | Sex | Age | IIF titer | IIF scores^1^ | Cumulative duration of medication(month)^2^ | Cure  (5 score)^3^ | Therapy maintenance  (10 score)^3^ | Death  (15 score)^3^ | Total score^4^ |
| --- | --- | --- | --- | --- | --- | --- | --- | --- | --- | --- | --- |
| BL151702 | EPPK1 | BP | female | 67 | 0 | 0 | 72 |  | 10 |  | 82 |
| BF10855 | EPPK1 | BP | male | 69 | 160 | 5 | 60 | 5 |  |  | 70 |
| BL163476 | EPPK1 | BP | male | 62 | 10 | 1 | 15 | 5 |  |  | 21 |
| BL181772 | KRT5 | BP | male | 62 | 0 | 0 | 50 | 5 |  |  | 55 |
| BL182013 | #N/A | BP | female | 75 | 640 | 7 | 6 | 5 |  |  | 18 |
| BL182867 | PLEC | BP | female | 69 | 10 | 1 | 24 | 5 |  |  | 30 |
| BL183066 | #N/A | BP | female | 45 | 10 | 1 | 10 | 5 |  |  | 16 |
| BL183360 | #N/A | BP | male | 71 | 160 | 5 | 4 | 5 |  |  | 14 |
| BL190072 | EPPK1 | BP | male | 77 | 320 | 6 | 18 |  |  | 15 | 39 |
| HLA3094 | EPPK1 | BP | male | 78 | 640 | 7 | 18 |  |  | 15 | 40 |
| HLA3866 | EPPK1,LAMB3,LAMC1 | BP | female | 63 | 320 | 6 | 84 |  | 10 |  | 100 |
| HLA4375 | EPPK1 | BP | male | 64 | 640 | 7 | 36 | 5 |  |  | 48 |
| HLA4666 | EPPK1,JUP,LAMC2 | BP | male | 54 | 1280 | 8 | 36 |  |  | 15 | 59 |
| BL151805 | EPPK1,LAMA3,LAMB3,PLEC | DH | female | 47 | 20 | 2 | 96 |  | 10 |  | 108 |
| HLA3207 | #N/A | BP | male | 61 | 160 | 5 | 10 | 5 |  |  | 20 |
| BL180560 | #N/A | BP | male | 67 | 10 | 1 | 30 | 5 |  |  | 36 |
| BL193512 | #N/A | DH | female | 52 | 0 | 0 | 6 |  | 10 |  | 16 |
| BL193798 | PLEC | DH | female | 20 | 160 | 5 | 48 |  | 10 |  | 63 |
| BL194176 | LAMC2 | DH | male | 38 | 160 | 5 | 48 |  | 10 |  | 63 |
| BL194752 | LAMA3,LAMC2 | DH | female | 29 | 160 | 5 | 48 |  | 10 |  | 63 |
| HLA3477 | EPPK1,ERBIN | DH | male | 26 | 40 | 3 | 84 |  | 10 |  | 97 |
| BL153331 | EPPK1,ITGB4 | LigA | female | 75 | 20 | 2 | 36 |  |  | 15 | 53 |
| BF12541 | PLEC | BP | male | 45 | 10 | 1 | 30 | 5 |  |  | 36 |
| BL184176 | #N/A | LigA | female | 86 | 0 | 0 | 10 | 5 |  |  | 15 |
| BL184219 | #N/A | LigA | female | 42 | 160 | 5 | 12 | 5 |  |  | 22 |
| BL194226 | #N/A | LigA | male | 41 | 160 | 5 | 10 | 5 |  |  | 20 |
| HLA4547 | EPPK1,ERBIN | LigA | female | 27 | 10 | 1 | 78 |  | 10 |  | 89 |
| IgA0011 | EPPK1 | LigA | male | 7 | 320 | 6 | 120 |  | 10 |  | 136 |
| BL172519 | LAMA3 | BP | female | 75 | 1280 | 8 | 48 |  |  | 15 | 71 |
| BL151713 | #N/A | BP | male | 79 | 0 | 0 | 36 |  |  | 15 | 51 |
| HLA3795 | LAMC2,DST | BP | female | 87 | 1280 | 8 | 48 |  | 10 |  | 66 |
| HLA3419 | #N/A | BP | male | 59 | 1280 | 8 | 18 | 5 |  |  | 31 |
| BL151846 | #N/A | BP | male | 47 | 0 | 0 | 6 | 5 |  |  | 11 |
| BL152117 | #N/A | BP | male | 83 | 40 | 3 | 10 | 5 |  |  | 18 |
| BL152512 | #N/A | BP | male | 43 | 0 | 0 | 6 | 5 |  |  | 11 |
| BL152572 | #N/A | BP | female | 80 | 0 | 0 | 6 | 5 |  |  | 11 |
| HLA4331;BL173402 | ERBIN | BP | male | 45 | 0 | 0 | 48 | 5 |  |  | 53 |
| HLA5287 | ITGA3 | BP | male | 63 | 640 | 7 | 18 | 5 |  |  | 30 |
| BF11815 | #N/A | BP | male | 88 | 1280 | 8 | 24 | 5 |  |  | 37 |
| BL183243 | #N/A | BP | male | 82 | 0 | 0 | 6 |  |  | 15 | 21 |
| HLA0910 | #N/A | BP | female | 26 | 0 | 0 | 10 | 5 |  |  | 15 |
| BL160306 | PLEC | BP | male | 63 | 640 | 7 | 24 |  |  | 15 | 46 |
| HLA2012 | #N/A | BP | female | 63 | 320 | 6 | 12 | 5 |  |  | 23 |
| BL183250 | PLEC,LAMC1 | LigA | female | 52 | 0 | 7 | 12 | 5 |  |  | 24 |
| BF10844 | #N/A | BP | male | 68 | 160 | 5 | - | - | - | - | 5 |
| BF11458 | DST | BP | male | 73 | 10 | 1 |  | 5 |  |  | 6 |
| BL173784 | PLEC | BP | female | 80 | 640 | 7 | 12 | 5 |  |  | 24 |
| BF11536 | #N/A | BP | male | 61 | 1280 | 8 | - | - | - | - | 8 |
| SGD1469 | EPPK1 | LigA | female | 0 | 320 | 6 | 13 | 5 |  |  | 24 |
| BF12027 | #N/A | BP | female | 50 | 0 | 0 | 12 | 5 |  |  | 17 |
| BF12048 | DST | BP | female | 62 | 160 | 5 | 84 |  | 10 |  | 99 |
| HLA2095 | COL17A1 | BP | male | 58 | 320 | 6 | 13 | 5 |  |  | 24 |
| BF12496 | #N/A | BP | female | 70 | 1280 | 8 | - | - | - | - | 8 |
| BL160577 | KRT14,LAMC1 | BP | female | 48 | 640 | 7 | 84 |  | 10 |  | 101 |
| HLA3873 | LAMC2 | BP | female | 24 | 10 | 1 | 32 | 5 |  |  | 38 |
| BF12693 | #N/A | BP | female | 73 | 0 | 0 | 36 | 5 |  |  | 41 |
| BF13099 | #N/A | BP | male | 59 | 40 | 3 | 8 | 5 |  |  | 16 |
| BF13319 | #N/A | BP | female | 47 | 1280 | 8 | - | - | - | - | 8 |
| BL151654;HLA4102 | ITGB4,JUP,ITGB4 | BP | female | 81 | - | 6 | 60 |  |  | 15 | 81 |
| BL151969 | #N/A | BP | female | 59 | 10 | 1 | 6 | 5 |  |  | 12 |
| BL152307 | #N/A | BP | male | 59 | 10 | 1 | - | - | - | - | 1 |
| BL171561;BF12624 | ITGB4 | BP | female | 71 | 0 | 0 | 36 | 5 |  |  | 41 |
| BL160269 | #N/A | BP | female | 78 | 10 | 1 | - | 5 | - | - | 6 |
| BF11620 | KRT14,LAMC1 | EBA | female | 48 | 640 | 7 | 84 |  | 10 |  | 101 |
| BL163153 | PLEC | BP | female | 47 | 0 | 0 | 84 |  | 10 |  | 94 |
| HLA5199 | LAMA3,LAMC2 | BP | female | 75 | 160 | 5 | 48 |  | 10 |  | 63 |
| BL170425 | #N/A | BP | male | 75 | 10 | 1 | - | 5 | - | - | 6 |
| BL170497 | #N/A | BP | male | 57 | 40 | 3 | 60 | 5 |  |  | 68 |
| BL170660 | PLEC | BP | male | 52 | 1280 | 8 | 27 | 5 |  |  | 40 |
| BF12116;BL161036 | PLEC | BP | male | 87 | 40 | 3 | 84 |  |  | 15 | 102 |
| BL172163 | #N/A | BP | male | 83 | 160 | 5 | 1 |  |  | 15 | 21 |
| BL163590 | PLEC | BP | male | 51 | 80 | 4 | 84 |  | 10 |  | 98 |
| BL173327 | #N/A | BP | male | 64 | - | 5 | 36 | 5 |  |  | 46 |
| HLA3867 | LAMA3 | BP | male | 50 | 160 | 5 | 10 | 5 |  |  | 20 |
| BL173540 | #N/A | BP | male | 71 | 1280 | 8 |  | 60 | 10 |  | 78 |
| BL173750 | #N/A | BP | male | 69 | 160 | 5 | 24 | 5 |  |  | 34 |
| BL181456 | DST | BP | female | 64 | 10 | 1 | 10 | 5 |  |  | 16 |
| BL181524 | #N/A | BP | male | 72 | 1280 | 8 | 6 | 5 |  |  | 19 |
| HLA2683 | #N/A | BP | male | 58 | 160 | 5 | 48 | 5 |  |  | 58 |
| HLA3012 | #N/A | BP | female | 68 | 640 | 7 | 48 | 5 |  |  | 60 |
| HLA3017 | #N/A | BP | female | 63 | 640 | 7 | - | - | - | - | 7 |
| HLA6248 | ITGA3 | BP | male | 26 | 1280 | 8 | 10 | 5 |  |  | 23 |
| BL191450 | #N/A | DH | female | 36 | 10 | 1 | 60 | 5 |  |  | 66 |
| BL151736 | JUP | BP | female | 57 | 10 | 1 | 60 | 5 |  |  | 66 |
| HLA3158 | #N/A | BP | male | 70 | 1280 | 8 | 8 | 5 |  |  | 21 |
| IgA0006 | #N/A | LigA | female | 25 | 10 | 1 | - | - | - | - | 1 |
| HLA2599 | #N/A | LigA | male | 60 | 10 | 1 | 12 | 5 | - | - | 18 |
| HLA5036 | PLEC | BP | male | 54 | 0 | 0 | 8 | 5 |  |  | 13 |
| HLA2982 | #N/A | BP | female | 70 | 1280 | 8 | 72 |  |  | 15 | 95 |
| HLA4622 | LAMA3 | BP | female | 73 | 10 | 1 | 72 |  | 10 |  | 83 |
| HLA3447 | #N/A | BP | male | 82 | 1280 | 8 | 3 |  |  | 15 | 26 |
| HLA3493 | #N/A | LigA | male | 61 | 0 | 0 | 6 | 5 |  |  | 11 |
| HLA3515 | #N/A | LigA | female | 81 | 10 | 1 | 10 | 5 |  |  | 16 |
| HLA3702 | LAMB3 | LigA | female | 55 | 0 | 0 | 72 |  | 10 |  | 82 |
| HLA3751 | #N/A | BP | female | 41 | 40 | 3 | 3 | 5 |  |  | 11 |
| BL171838 | EPPK1 | DH | male | 28 | 1280 | 8 | 72 |  | 10 |  | 90 |
| HLA3898 | #N/A | LigA | female | 58 | 0 | 0 | 3 | 5 |  |  | 8 |
| HLA3974 | #N/A | BP | female | 78 | 1280 | 8 | 2 |  |  | 15 | 25 |
| BL151720 | PLEC | BP | female | 81 | 320 | 6 | 24 |  |  | 15 | 45 |
| BL160210 | COL17A1 | BP | female | 69 | 1280 | 8 | 6 | 5 |  |  | 19 |
| HLA4041 | ITGA6 | BP | male | 85 | 0 | 0 | 72 |  | 10 |  | 82 |
| HLA4066 | #N/A | BP | female | 89 | 1280 | 8 | 3 |  |  | 15 | 26 |
| BL162288;BF0012012 | #N/A | EBA | male | 66 | 10 | 1 | 8 | 5 |  |  | 14 |
| HLA1999 | #N/A | BP | male | 72 | 640 | 7 | 36 | 5 |  |  | 48 |
| BL142610 | #N/A | DH | male | 93 | 10 | 1 | - | 5 | - | - | 6 |
| BL151876 | #N/A | DH | male | 49 | 0 | 0 | 6 | 5 |  |  | 11 |
| BL152024 | PLEC | DH | male | 48 | 0 | 0 | 84 |  | 10 |  | 94 |
| BL153381 | #N/A | DH | female | 49 | 40 | 3 | 6 | 5 |  |  | 14 |
| BL170065 | #N/A | DH | female | 63 | 0 | 0 | 36 |  |  |  | 36 |
| BL172140 | #N/A | DH | male | 40 | 0 | 0 | 6 | 5 |  |  | 11 |
| BL173442 | #N/A | DH | male | 70 | 0 | 0 | - | - | - | - | 0 |
| BL173838 | ITGB4 | DH | male | 27 | 10 | 1 | 48 | 5 |  |  | 54 |
| BL174993 | LAMC2 | DH | female | 37 | 160 | 5 | 72 |  | 10 |  | 87 |
| BL180557 | ITGB4 | DH | female | 66 | 320 | 6 | 12 | 5 |  |  | 23 |
| BL180861 | ITGA3,JUP | DH | male | 21 | 0 | 0 | 60 |  | 10 |  | 70 |
| BL181095 | PLEC | DH | female | 59 | 0 | 0 | 48 |  | 10 |  | 58 |
| BL181805 | #N/A | DH | male | 31 | 0 | 0 | - | - | - | - | 0 |
| BL181830 | #N/A | DH | male | 35 | 0 | 0 | - | - | - | - | 0 |
| BL182547 | PLEC | DH | female | 43 | 160 | 5 | 10 | 5 |  |  | 20 |
| BL183482 | #N/A | DH | male | 56 | 10 | 1 | - | 5 | - | - | 6 |
| BL184618 | #N/A | DH | female | 61 | 10 | 1 | - | 5 | - | - | 6 |
| BL190152 | JUP | DH | male | 13 | 0 | 0 | 6 | 5 |  |  | 11 |
| HLA4332 | #N/A | EBA | male | 55 | 10 | 1 | - | 5 | - | - | 6 |
| HLA4502 | #N/A | BP | male | 63 | 40 | 3 | 36 | 5 |  |  | 44 |
| HLA1574 | DST,ITGA6 | DH | male | 27 | 40 | 3 | 84 |  | 10 |  | 97 |
| HLA4714 | DST | BP | female | 51 | 1280 | 8 | 3 | 5 |  |  | 16 |
| HLA4817 | #N/A | LigA | female | 52 | 0 | 0 | 6 | 5 |  |  | 11 |
| HLA5255 | LAMA3 | DH | male | 17 | 0 | 0 | 6 | 5 |  |  | 11 |
| HLA4835 | #N/A | LigA | female | 6 | 0 | 0 | 6 | 5 |  |  | 11 |
| HLA4985 | ERBIN | BP | male | 82 | 40 | 3 | 24 |  |  | 15 | 42 |
| HLA5991 | PLEC,LAMB3 | DH | male | 25 | 160 | 5 | 48 |  | 10 |  | 63 |
| HLA5109 | #N/A | DH | female | 63 | 10 | 1 | - | - | - | - | 1 |
| HLA6183 | #N/A | DH | female | 62 | 0 | 0 | - | - | - | - | 0 |
| HLA5201 | #N/A | DH | male | 65 | 10 | 1 | - | - | - | - | 1 |
| HLA5358 | #N/A | LigA | female | 43 | 0 | 0 | 16 | 5 |  |  | 21 |
| HLA5412 | #N/A | LigA | female | 80 | 10 | 1 | - | 5 | - | - | 6 |
| BF0010589 | LAMA3,PLEC | EBA | female | 48 | 640 | 7 | 72 | 5 |  |  | 84 |
| BF13074 | LAMC2 | BP | female | 65 | 640 | 7 | 72 |  | 10 |  | 89 |
| BL151683 | PLEC | EBA | male | 72 | 0 | 0 | 12 | 5 |  |  | 17 |
| BL162003 | ITGA6 | EBA | male | 64 | 320 | 6 | 84 |  | 10 |  | 100 |
| BF11482 | PLEC | BP | male | 71 | 10 | 1 | 6 | 5 |  |  | 12 |
| BL173874 | LAMB3 | EBA | male | 67 | 10 | 1 | 6 | 5 |  |  | 12 |
| BL190073 | DST | EBA | male | 72 | 160 | 5 | 4 | 5 |  |  | 14 |
| BL191060 | #N/A | EBA | male | 31 | 1280 | 8 | - | - | - | - | 8 |
| BL192225 | PLEC | EBA | female | 48 | 160 | 5 | 48 |  | 10 |  | 63 |
| BL192983 | #N/A | EBA | female | 25 | 0 | 0 | 36 | 5 |  |  | 41 |
| BL193577 | DST | EBA | male | 63 | 320 | 6 | 36 |  | 10 |  | 52 |
| BL194161 | JUP,LAMB3 | EBA | female | 69 | 160 | 5 | 120 | 5 |  |  | 130 |
| HLA3273 | PLEC | EBA | female | 8 | 320 | 6 | 48 | 5 |  |  | 59 |
| HLA4068 | PLEC | EBA | female | 60 | 160 | 5 | - | - | - | - | 5 |
| HLA5740 | #N/A | DH | male | 73 | 0 | 0 | 32 |  | 10 |  | 42 |
| HLA5786 | #N/A | DH | male | 47 | 0 | 0 | 48 |  | 10 |  | 58 |
| HLA5869 | #N/A | BP | male | 56 | 0 | 0 | 24 | 5 |  |  | 29 |
| BL140869 | #N/A | LigA | male | 26 | 0 | 0 | - | 5 |  |  | 5 |
| BL141074 | #N/A | LigA | female | 43 | 10 | 1 | - | 5 |  |  | 6 |
| BL141814 | #N/A | LigA | female | 27 | 10 | 1 | - | 5 | - | - | 6 |
| BL151312 | #N/A | LigA | female | 42 | 10 | 1 | - | 5 | - | - | 6 |
| BL151316 | ERBIN | LigA | male | 52 | 0 | 0 | 10 | 5 |  |  | 15 |
| BL161331 | #N/A | LigA | male | 26 | 40 | 3 | 12 | 5 |  |  | 20 |
| BL161493 | #N/A | LigA | female | 55 | 160 | 5 | 15 | 5 |  |  | 25 |
| BL161897 | ITGA3 | LigA | female | 18 | 40 | 3 | 12 | 5 |  |  | 20 |
| BL171061 | #N/A | LigA | female | 66 | 0 | 0 | - | - | - | - | 0 |
| BL173381 | #N/A | LigA | male | 29 | 160 | 5 | 6 | 5 |  |  | 16 |
| HLA6113 | #N/A | DH | male | 15 | 10 | 1 | - | 5 | - | - | 6 |
| HLA6110 | #N/A | DH | male | 53 | 40 | 3 | - | - | - | - | 3 |
| HLA6143 | #N/A | EBA | female | 70 | 10 | 1 | 6 | 5 | - | - | 12 |
| HLA6182 | DST,LAMB3,ITGA6 | BP | male | 23 | 1280 | 8 | 48 |  | 10 |  | 66 |
| HLA6218 | #N/A | BP | male | 26 | 160 | 5 | 24 | 5 |  |  | 34 |
| HLA6230 | #N/A | DH | male | 66 | 10 | 1 | - | 5 | - | - | 6 |
| HLA6239 | #N/A | EBA | female | 17 | 640 | 7 | 24 |  | 10 |  | 41 |
| HLA4019 | KRT14 | LigA | male | 73 | 160 | 5 | 6 | 5 |  |  | 16 |
| HLA6244 | #N/A | DH | male | 49 | 10 | 1 | - | 5 | - | - | 6 |
| HLA6269 | #N/A | LigA | male | 7 | 40 | 3 | 36 |  | 10 |  | 49 |
| HLA4924 | COL17A1 | LigA | male | 53 | 320 | 6 | 60 |  | 10 |  | 76 |
| HLA0011 | #N/A | DH | male | 37 | 10 | 1 | - | 5 | - | - | 6 |
| HLA0630 | #N/A | DH | male | 12 | 10 | 1 | - | 5 | - | - | 6 |
| HLA6112 | LAMA3 | LigA | male | 40 | 160 | 5 | 12 | 5 |  |  | 22 |
| HLA6188 | LAMC2 | LigA | female | 4 | 40 | 3 | 72 |  | 10 |  | 85 |
| HLA0604 | DST,,ITGA6 | LigA | female | 1 | 40 | 3 | 108 |  | 10 |  | 121 |
| HLA6274 | LAMA3 | LigA | male | 1 | 40 | 3 | 48 | 5 |  |  | 56 |
| IgA0001 | #N/A | LigA | male | 60 | 10 | 1 | - | - | - | - | 1 |
| IgA0002 | LAMC2 | LigA | male | 25 | - | 8 | 120 | 5 |  |  | 133 |
| IgA0003 | DST | LigA | female | 73 | - | - | 120 | 5 |  |  | 125 |
| IgA0005 | DST | LigA | male | 28 | 10 | 1 | 120 | 5 | - | - | 126 |
| HLA0881 | #N/A | LigA | female | 61 | 10 | 1 | - | - | - | - | 1 |
| IgA0007 | ITGB4 | LigA | male | 75 | - | 8 | 120 | - | - | 15 | 143 |
| IgA0008 | JUP | LigA | male | 31 | - | 8 | 36 | 5 | - | - | 49 |
| IgA0009 | COL17A1,KRT5 | LigA | female | 5 | - | 8 | 60 | 5 | - | - | 73 |
| IgA0010 | #N/A | LigA | male | 4 | 10 | 1 | - | - | - | - | 1 |
| IgA0012 | #N/A | LigA | male | 78 | 10 | 1 | - | - | - | - | 1 |
| IgA0047 | #N/A | LigA | female | 57 | 10 | 1 | - | - | - | - | 1 |
| IgA0057 | ITGB4,DST,LAMC1 | LigA | male | 31 | 0 | 0 | 84 |  | 10 |  | 94 |
| SGD0884 | ITGB4 | LigA | female | 32 | - | 8 | - | - | - | - | 8 |
| SGD1468 | COL17A1,KRT5 | LigA | female | 32 | - | 8 | - | - | - | - | 8 |
| SGD1470 | #N/A | LigA | female | 45 | 10 | 1 | 10 | 5 | - | - | 16 |
| SGD1471 | LAMA3 | LigA | female | 53 | 320 | 6 | 120 | 5 |  |  | 131 |
| BF0013786 | #N/A | BP | female | 79 | 40 | 3 | - | - | - | - | 3 |
| BL151713 | #N/A | BP | female | 72 | 0 | 0 | - | - | - | - | 0 |
| BL151826 | #N/A | BP | male | 91 | 320 | 6 | - | - | - | - | 6 |
| BL161036 | #N/A | BP | male | 62 | 0 | 0 | - | - | - | - | 0 |
| BL143344 | #N/A | EBA | female | 18 | 160 | 5 | - | - | - | - | 5 |

Explanation: The calculation of the Total score is equal to the sum of the scores obtained from three aspects: the IIF score^1^, the cumulative duration of medication (in months)^2^, and the disease outcome^3^ (which includes three categories: cure, Therapy maintenance, and Death).
